# Characterization of patients with major psychiatric disorders with AMPA receptor positron emission tomography

**DOI:** 10.1038/s41380-024-02785-1

**Published:** 2024-10-15

**Authors:** Mai Hatano, Waki Nakajima, Hideaki Tani, Hiroyuki Uchida, Tomoyuki Miyazaki, Tetsu Arisawa, Yuuki Takada, Sakiko Tsugawa, Akane Sano, Kotaro Nakano, Tsuyoshi Eiro, Hiroki Abe, Akira Suda, Takeshi Asami, Akitoyo Hishimoto, Nobuhiro Nagai, Teruki Koizumi, Shinichiro Nakajima, Shunya Kurokawa, Yohei Ohtani, Kie Takahashi, Yuhei Kikuchi, Taisuke Yatomi, Shiori Honda, Masahiro Jinzaki, Yoji Hirano, Ryo Mitoma, Shunsuke Tamura, Shingo Baba, Osamu Togao, Hirotaka Kosaka, Hidehiko Okazawa, Yuichi Kimura, Masaru Mimura, Takuya Takahashi

**Affiliations:** 1 Department of Physiology, Yokohama City University Graduate School of Medicine, Yokohama, Japan; 2 Department of Neuropsychiatry, Keio University School of Medicine, Tokyo, Japan; 3 Center for Promotion of Research and Industry-Academic Collaboration, Yokohama City University, Yokohama, Japan; 4 Radioisotope Research Center, Yokohama City University Graduate School of Medicine, Yokohama, Japan; 5 Department of Psychiatry, Yokohama City University Graduate School of Medicine, Yokohama, Japan; 6 Department of Psychiatry, Kobe University Graduate School of Medicine, Kobe, Japan; 7 Department of Radiology, Keio University School of Medicine, Tokyo, Japan; 8 Department of Psychiatry, Division of Clinical Neuroscience, Faculty of Medicine, University of Miyazaki, Miyazaki, Japan; 9 Department of Neuropsychiatry, Graduate School of Medical Sciences, Kyusyu University, Fukuoka, Japan; 10 Institute of Industrial Science, The University of Tokyo, Tokyo, Japan; 11 Department of Clinical Radiology, Graduate School of Medical Sciences, Kyusyu University, Fukuoka, Japan; 12 Department of Psychiatry, Faculty of Medical Sciences, University of Fukui, Fukui, Japan; 13 Biomedical Imaging Research Center, University of Fukui, Fukui, Japan; 14 Faculty of Informatics, Cyber Informatics Research Institute, Kindai University, Higashi-Osaka, Japan; 15 The International Research Center for Neurointelligence, Institutes for Advanced Study, University of Tokyo, Tokyo, Japan

**Keywords:** Neuroscience, Psychiatric disorders

## Abstract

Synaptic phenotypes in living patients with psychiatric disorders are poorly characterized. Excitatory glutamate α-amino-3-hydroxy-5-methyl-4-isoxazole propionic acid receptor (AMPAR) is a fundamental component for neurotransmission. We recently developed a positron emission tomography (PET) tracer for AMPAR, [^11^C]K-2, the first technology to visualize and quantify AMPARs density in living human brain. In this study, we characterized patients with major psychiatric disorders with [^11^C]K-2. One hundred forty-nine patients with psychiatric disorders (schizophrenia, n = 42; bipolar disorder, n = 37; depression, n = 35; and autism spectrum disorder, n = 35) and 70 healthy participants underwent a PET scan with [^11^C]K-2 for measurement of AMPAR density. We detected brain regions that showed correlation between AMPAR density and symptomatology scores in each of four disorders. We also found brain areas with significant differences in AMPAR density between patients with each psychiatric disorder and healthy participants. Some of these areas were observed across diseases, indicating that these are commonly affected areas throughout psychiatric disorders. Schizophrenia, bipolar disorder, depression, and autism spectrum disorder are uniquely characterized by AMPAR distribution patterns. Our approach to psychiatric disorders using [^11^C]K-2 can elucidate the biological mechanisms across diseases and pave the way to develop novel diagnostics and therapeutics based on the synapse physiology.

## Introduction

Psychiatric disorders such as schizophrenia, bipolar disorder, depression, and autism spectrum disorder (ASD) are common serious diseases which impair social functioning and lower quality of life [1] on an individual level as well as a significant economic loss on a societal level [2, 3]. Despite the grave consequences caused by these illnesses, the biological bases, especially synaptic and circuit substrates, underlying psychiatric disorders still remain elucidated. Due to the lack of biological basis, diagnosis and disease classification of psychiatric disorders are currently based on clinical symptoms [4]. However, patients with different “diagnoses” often show the same clinical symptoms [5–7], while those with the same “diagnosis” are sometimes assumed to have different biological backgrounds [8] raising questions about the validity of the current symptom-based diagnostic system. Thus, it is crucial to establish a new disease classification based on biological characteristics so as to elucidate the pathophysiological mechanisms of psychiatric disorders and develop novel diagnostics and therapeutics. To achieve this goal, a cross-“disease” research with biological modality is needed.

Recent findings from animal models, genetic studies, and post-mortem brain studies suggest that one of the features of psychiatric disorders are considered to be “synapse diseases” [9–14] in which malfunction of synapses can underlie psychiatric disorders. Thus, biological basis of psychiatric disorders could theoretically be identified by monitoring synaptic phenotypes in patients with psychiatric disorders, which however have been poorly characterized due to technical limitations as described below.

Glutamate synapses play essential roles in neuronal function in which fast transmission is mainly mediated by α-amino-3-hydroxy-5-methyl-4-isoxazole propionic acid receptors (AMPARs) [15–22]. Thus, AMPAR is a principal component of neurotransmission [23]. Therefore, a technology for visualization and quantification of AMPARs in the living human brain and subsequent synaptic characterization of patients with psychiatric disorders has long been desired. We have recently developed the positron emission tomography (PET) tracer for AMPARs, [^11^C]K-2, the first technology to visualize and quantify the density of AMPARs in the living human brain [24]. [^11^C]K-2 indicated a good reversible kinetics that is appropriate to quantify AMPA density using PET. We exhibited that Standardized uptake value ratio (SUVR) using the white matter as a reference (quantitative value of absolute AMPARs density in [^11^C]K-2) showed strong significant positive correlations with local AMPAR density in resected brain tissues of patients with mesial temporal lobe epilepsy [24], and therefore, SUVR obtained from [^11^C]K-2 PET scan can be used for clinical investigation. Further, PET image with [^11^C]K-2 depicts cell surface AMPARs, a functionally crucial fraction of AMPARs [25]. Here, we PET-scanned 149 patients with psychiatric disorders, using [^11^C]K-2, that included schizophrenia (42 patients), bipolar disorder (37 patients), depression (35 patients), and ASD (35 patients). In summary, here we reported synaptic phenotypes of these psychiatric disorders. By using SUVR with the whole brain as the reference region, we characterized changes in the balance of the density of AMPARs among multiple brain areas, potentially providing biological features of major psychiatric disorders.

## Patients and methods

### Ethics statement

This study comprised five clinical studies that were registered under the following IDs: UMIN000025132, jRCTs031190197, jRCTs031190150, jRCTs031190149, and jRCTs031200083 which targeted multiple diagnoses, schizophrenia, bipolar disorder and depression, ASD, and healthy participants, respectively. CONSORT chart is provided in the Supplementary Figs. 1–5. All studies were approved by Yokohama City University Human Investigation Committee and Yokohama City University Certified Institutional Review Board in accordance with the Ethical guidelines for medical and health research involving human participants by the Japan Ministry of Health, Labour and Welfare and the Clinical Trials Act in Japan. Data from these five studies were combined and analysed with the approval of the Yokohama City University Human Investigation Committee (trial registry number jRCT1030220303). This study was conducted at Yokohama City University Hospital, Keio University Hospital, Kyushu University Hospital, and University of Fukui Hospital between August 2016 and April 2022. All participants provided written informed consent after receiving detailed information about the protocol. Clinical assessments were performed by one of the trained investigators who was blind to the PET and magnetic resonance imaging (MRI) data.

### Participants

#### Patients with schizophrenia

Selection criteria for participants with four psychiatric disorders and healthy participants are detailed in the Supplementary methods. Briefly, in the first study (UMIN000025132) the inclusion criteria were: male in- and outpatients 30–49 years of age; patients who met the Diagnostic and Statistical Manual of Mental Disorders Fourth Edition (DSM-IV) criteria for schizophrenia [26], using the structured clinical interview for DSM-IV (SCID-I/DSM-IV) [27]. In the second study (jRCTs031190197), the inclusion criteria were the same as those in the first study, other than the age range (i.e., 20–59 years) and sex (i.e., both men and women were included). The demographic and clinical characteristics of the 42 patients (30 men) included in the PET analysis were as follows: age, 38.9 ± 9.1 years; illness duration, 13.5 ± 9.3 years; scores in the Positive and Negative Syndrome Scale (PANSS) [28], 75.7 ± 24.4 (total), 18.1 ± 7.1 (positive symptoms), 20.7 ± 6.7 (negative symptoms) and 37.1 ± 13.4 (general psychopathology) as shown in the Supplementary methods and Supplementary Table 1.

#### Patients with bipolar disorder

Briefly, in the first study (UMIN000025132) the inclusion criteria were: male in- and outpatients 30–49 years of age; and patients who met the DSM-IV criteria for bipolar disorder using the SCID-I/DSM-IV [27]. In the second study (jRCTs031190150), the inclusion criteria were the same as those in the first study, other than the age range (i.e., 20–59 years) and sex (i.e., both men and women were included). The demographic and clinical characteristics of the 37 patients (22 men) with bipolar disorder included in this analysis are as follows: age, 41.8 ± 8.2 years; illness duration, 15.4 ± 8.1 years; total score in the 17-item Hamilton Depression Rating Scale (HAM-D) [29], 6.2 ± 5.3, the total score in the Young Mania Rating Scale (YMRS) [30], 7.1 ± 7.1 as shown in the Supplementary methods and Supplementary Table 2.

#### Patients with depression

Briefly, in the first study (UMIN000025132) the inclusion criteria were: male in- and outpatients 30–49 years of age; and patients who met the DSM-IV criteria for major depressive disorder using the SCID-I/DSM-IV [27]. In the second study (jRCTs031190150), the inclusion criteria were the same as those in the first study, other than the age range (i.e., 20–59 years) and sex (i.e., both men and women were included). The demographic and clinical characteristics of the 35 patients (27 men) included in this analysis are as follows: age, 43.0 ± 7.4 years; illness duration, 7.9 ± 7.4 years; total score in the HAM-D, 10.3 ± 7.4 as shown in the Supplementary methods and Supplementary Table 3.

#### Patients with autism spectrum disorder (ASD)

Briefly, in the first study (UMIN000025132) the inclusion criteria were: male in- and outpatients 30–49 years of age; and patients who met the DSM-5 ASD criteria. In the second study (jRCTs031190149), the inclusion criteria were the same as those in the first study, other than the age range (i.e., 20–59 years), sex (i.e., both men and women were included), and intellectual performance (i.e., full-scale intelligence quotient [FIQ] ≥ 70 according to Wechsler Adult Intelligence Scale third edition [WAIS-III] or Wechsler Adult Intelligence Scale fourth edition [WAIS-IV]). The demographic and clinical characteristics of the 35 patients (28 men) in this analysis are as follows: age, 33.1 ± 7.5 years; illness duration, 19.4 ± 12.5 years; Autism Diagnostic Observation Schedule second edition (ADOS-2) [31] calibrated severity score (CSS), 7.3 ± 2.2 as shown in the Supplementary methods and Supplementary Table 4.

#### Healthy participants

In the first study (UMIN000025132) the inclusion criteria were: healthy male participants who were 30–79 years of age and did not fulfill any diagnostic criteria for psychiatric conditions according to the DSM-IV [26] using the SCID-I/DSM-IV [27]. Among them, age-matched (i.e., 30–59 years) healthy participants were included. In the second study (jRCTs031200083), the selection criteria were the same as those in the first study, other than the age range (i.e., 20–49 years) and sex (i.e., both men and women were included). The demographic characteristics of the participants are shown in the Supplementary methods.

##### PET and MRI imaging

The participants underwent a PET scan with [^11^C]K-2 and an MRI scan. Details of imaging settings and procedures are detailed in the Supplementary methods. Briefly, [^11^C]K-2 was synthesized locally at Yokohama City University Hospital, Keio University Hospital Kyushu University Hospital, and University of Fukui Hospital in accordance with GMP ordinance. On the day of the PET scan, the patients with psychiatric disorder received the clinical assessments. These assessments were performed by one of the trained investigators who was blind to the PET and MRI data.

##### Quantification of AMPA receptor density with PET

To quantify receptor density, a non-displaceable binding potential (*BP*_ND_) that is a quantitative index of receptor density is commonly utilized.

We computed *BP*_ND_ using LGA with reference region (Logan Graphical Analysis [32]). In this study, we introduced a reference region that is the white matter where no expression of AMPARs was detected [24]. LGA is a widely used graphical analysis that uses linear regression to analyze pharmacokinetics of tracers using tissue time activity curves (tTAC; time course of radioactivity in the dynamic scan during 60 min). The plots of relationship between an integrated tTAC in each multiple brain regions and that in the white matter can be obtained by LGA. If these plots show good linear relation, [^11^C]K-2 achieves at an equilibrium state. Finally, we applied a linear regression 20 min after the administration of K-2 [24] and the slope of the plots provided us *BP*_ND_ in LGA [33, 34].

##### Standard uptake value ratio (SUVR)

SUVR images of [^11^C]K-2 were acquired by the followings: the radioactivity values of each brain regions are divided either by the radioactivity value of white matter during 30 min and 50 min after the tracer injection (SUVR_30–50 min_WM) or whole brain during the same period (SUVR_30–50 min_WB). SUVR_30–50 min_WM reflects “absolute” AMPARs density because of no expression of AMPARs in the white matter [24]. SUVR_30–50 min_WB shows “relative” AMPARs density among brain regions because the radioactivity values of each brain regions are normalized by the radioactivity of whole brain.

For calculation of *BP*_ND_, subjects needed to be performed a dynamic scan (scan time is 60 min). If we can prove that SUVR_30–50 min_WM is an appropriate surrogate marker of *BP*_ND_, we can acquire SUVR using a static PET scan (scan time is 20 min). It is more convenient to conduct the PET scan at the clinical site.

Therefore, we performed regression analysis between *BP*_ND_ and SUVR_30–50 min_WM-1 [24, 34].

To observe relative regional alterations in the [^11^C]K-2 signal in all participants, we used SUVR_30–50 min_WB. We also calculated correlation coefficients between SUVR_30–50 min_WB and *BP*_ND_.

The difference in Inter-individual between SUVR_30–50 min_WM and SUVR_30–50 min_WB　was examined using coefficient of variation (CV).

##### Normalizing PET images for SPM

A summed PET image at 30–50 min after injection of [^11^C]K-2 was obtained for each participant. SUVR_30-50 min_ images were normalized by white matter mean value (SUVR_30–50 min_WM) or whole brain mean value (SUVR_30–50 min_WB). We created the template for normalization from 3D-T1-weighted images of all participants using the high-dimensional nonlinear warping algorithm DARTEL [35]. SUVR_30–50 min_ images were spatially normalized into MNI standard space and preserved concentration to avoid volume effect using the template with SPM12.

##### Statistical analysis

The association between SUVR_30–50 min_WM or SUVR_30–50 min_WB values and clinical assessment scores of interests was assessed using multiple regression design implemented in SPM12. Statistical significance was set at *P* < 0.05 (peak-level uncorrected), false discovery rate (FDR) was corrected at *P* < 0.05 (cluster-level inference, FDRc) for multiple regression across all in-mask voxels. The cluster was extracted for correlation coefficient > 0.4. Comparison of SUVR_30-50 min_ values in patients with each psychiatric disorder (i.e., schizophrenia, bipolar disorder, depression, or ASD) and age-matched healthy participants was performed using two-sample t-test. Age-matched healthy participants were selected from the whole sample of healthy participants to correspond to the mean age of the patients. Therefore, data of the healthy participants in their 30 s, 40 s, and 50 s (n = 49; 30 men and 19 women; and mean age, 42.1 ± 7.1 years), those in their 20 s, 30 s, and 40 s (n = 66; 37 men and 29 women; and mean age, 35.6 ± 9.2 years), and those in their 20 s, 30 s, 40 s, and 50 s (n = 70; 41 men and 29 women; and mean age, 36.8 ± 10.2 years) were used for comparison to bipolar disorder (mean age, 41.8 ± 8.2 years; t-test, *P* = 0.8654; F-test, *P* = 0.3346, *F* = 1.345) and depression (mean age, 43.0 ± 7.4 years; t-test, *P* = 0.5893; F-test, *P* = 0.7557, *F* = 1.098), ASD (mean age, 33.1 ± 7.5 years; t-test, *P* = 0.1821; F-test, *P* = 0.2224, *F* = 1.470), schizophrenia (mean age, 38.9 ± 9.1 years; t-test, *P* = 0.2709; F-test, *P* = 0.4467, *F* = 1.249), respectively. Statistical significance was set at *P* < 0.05 (peak-level uncorrected), FDRc corrected at *P* < 0.05 for multiple comparisons across all in-mask voxels. To reduce the number of voxel-wise comparisons that were performed and avoid false positive findings, we used the mask of GM with image values of more than 10% in all above analyses, extracted from standard tissue probability maps equipped in SPM12. All analyses were adjusted for demographic covariates (age and sex) except 3D-rendered brains which were not adjusted covariates. Correlation analysis and t-test were conducted, and scatter plot was created using GraphPad Prism 8 (Graph Pad Software, Massachusetts, USA). All data fulfill the normality assumption.

##### Detection of commonly affected areas

In the participants with schizophrenia, bipolar disorder, and ASD, we created the binary images from reduction and increase regions (threshold is p < 0.05, FDRc) compared with age-matched healthy participants using SPM12. We identified the overlapped areas showing reduced and increased AMPAR densities, respectively, across these psychiatric disorders.

## Results

### Characteristics of [^11^C]K-2 in healthy participants

In order to characterize the distribution of AMPAR in brains of patients with psychiatric disorders, we first PET-scanned 70 healthy participants with [^11^C]K-2. As was previously observed with tissue time activity curves (tTACs) from multiple brain regions [24], we detected regional heterogeneity of the [^11^C]K-2 uptake with high AMPAR density in the cortex, putamen, and cerebellum (Supplementary Fig. 6A). To calculate the non-displaceable binding potential (*BP*_ND_; a quantitative index of AMPAR density utilized in [^11^C] K-2 imaging), Logan graphical analysis (LGA) was performed. LGA is a widely used graphical analysis that uses linear regression to analyze pharmacokinetics of tracers using tTACs. LGA using the white matter as a reference showed linearity in healthy participants, indicating the reversible binding kinetics of [^11^C]K-2 (Supplementary Fig. 6B). Based on LGA, we calculated *BP*_ND_ in each brain regions (see details in the method section) (Supplementary Table 5).

We first constructed the regional SUVR using white matter as a reference during 30–50 mins after the tracer injection (SUVR_30–50 min_WM) (Supplementary Fig. 6C and Supplementary Table 5). To confirm whether SUVR_30–50 min_WM is a surrogate for *BP*_ND,_ we performed regression analysis between *BP*_ND_ and SUVR_30–50min_WM-1. As was observed in healthy participants in the previous study [24], SUVR_30–50 min_WM-1 was compatible with *BP*_ND_ (Supplementary Fig. 6D). The linear relationship between *BP*_ND_ and SUVR_30–50 min_WM-1 was represented by Y = 0.9434*X +0.0182, indicating that SUVR_30–50 min_WM-1 is an appropriate surrogate outcome measure for absolute AMPAR density in healthy participants.

We also wondered if unbalanced AMPAR density among brain regions underlies pathogenesis of psychiatric disorder and aimed to examine relative regional changes of AMPAR density across the brain. We constructed the regional SUVR_30-50 min_ using whole brain as a reference (SUVR_30–50 min_WB) (Supplementary Fig. 6E and Supplementary Table 5). There exists a good linear relationship between *BP*_ND_ and SUVR_30–50min_WB (Supplementary Fig. 6F and Supplementary Table 6). These results indicate that SUVR_30–50 min_WB retain the differences of *BP*_ND_ among brain regions.

When we compared SUVR_30–50 min_WM with SUVR_30–50 min_WB, the coefficient of variation (CV) among healthy participants were lower with SUVR_30–50 min_WB than SUVR_30–50 min_WM, indicating that SUVR_30–50min_WB has less inter-individual variability and minimize influence of individual factor. (Supplementary Figs. 6C, E and Supplementary Table 7).

### Characteristics of [11C]K-2 in schizophrenia

We performed PET scanning of 42 patients with schizophrenia using [^11^C]K-2. As was previously observed in healthy participants, tTACs from multiple brain regions of [^11^C]K-2-injected patients with schizophrenia showed rapid radiotracer uptake in the brain and regional heterogeneity, with the lowest radioactivity observed in the white matter, where no AMPARs were detected (Supplementary Fig. 7A) [24]. LGA using the white matter as a reference showed linearity, indicating the reversible binding kinetics of [^11^C]K-2 in patients with schizophrenia as was observed in healthy participants (Supplementary Fig. 7B). As was observed in healthy participants, there exists a good linear relationship between *BP*_ND_ and SUVR_30–50 min_WM-1 (Supplementary Fig. 7C), indicating that SUVR_30–50 min_WM-1 is an appropriate surrogate outcome measure for absolute AMPAR density in patients with schizophrenia. To investigate relative regional alterations in the [^11^C]K-2 PET signal in patients with schizophrenia, we also constructed the SUVR_30–50 min_WB. There exists a good linear relationship between *BP*_ND_ and SUVR_30–50 min_WB (Supplementary Fig. 7D and Supplementary Table 8). The CV among patients with schizophrenia were lower with SUVR_30–50 min_WB than SUVR_30–50 min_WM, indicating that SUVR_30–50 min_WB has less inter-individual variability. (Supplementary Figs. 7E, F and Supplementary Table 7).

**Fig. 1:**
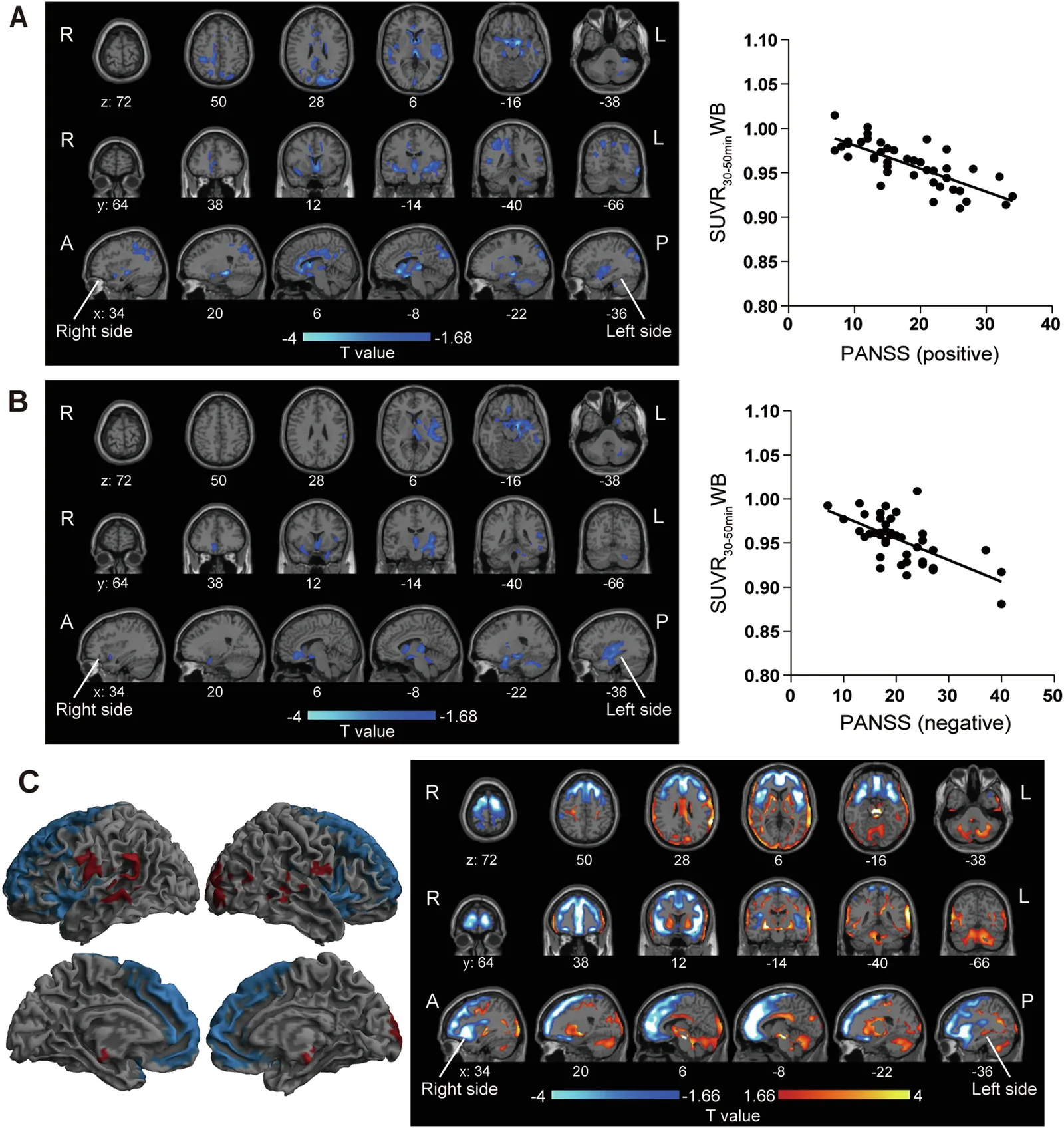
State regions and altered AMPAR distribution in patients with schizophrenia compared with healthy participants. **A** Brain regions showing a significant negative correlation between SUVR_30–50 min_WB and PANSS score for positive symptoms in patients with schizophrenia (*P* < 0.05, T < −1.68, one-tailed, FDRc). Significant clusters displayed on an axial, coronal and sagittal slices (Left), and scatter plot between averaged SUVR_30–50 min_WB in significant clusters and PANSS scores for positive symptoms (Right, two-tailed Pearson correlation analysis: correlation coefficient = −0.7514, *P* < 0.0001). **B** Brain regions showing a significant negative correlation between SUVR_30–50 min_WB and PANSS score for negative symptoms in patients with schizophrenia (*P* < 0.05, T < −1.68, one-tailed, FDRc). Significant clusters displayed on an axial, coronal and sagittal slices (Left), and scatter plot between averaged SUVR_30–50 min_WB in significant clusters and PANSS score for negative symptoms (Right, two-tailed Pearson correlation analysis: correlation coefficient = −0.6371, *P* < 0.0001). **C** Relative reduction (blue) and increase (red) of [^11^C]K-2 retention in patients with schizophrenia compared to healthy participants (*P* < 0.05, increase of [^11^C]K-2 retention: T > 1.66, reduction of [^11^C]K-2 retention: T < −1.66, one-tailed, FDRc). Significant clusters displayed on a 3D-rendered brain (binary image, Left), and axial, coronal and sagittal slices (T-map, Right). **A**, **B** and **C** (right panel) were adjusted for covariates (age, sex). **C** (left panel) was not adjusted for covariates.

We first performed regression analysis between SUVR_30–50 min_WM and the PANSS score (symptomatology score for schizophrenia). Voxel-wise analysis with SPM did not detect brain regions which exhibited correlation between them with statistical significance. Next, we examined whether the balance of AMPAR density is disrupted among brain regions, we used SUVR_30-50min_WB for the analysis. Voxel-wise analysis exhibited a significant negative correlation (*P* < 0.05, FDR corrected cluster-level inference (FDRc)) between the SUVR_30–50 min_WB and the PANSS score for positive symptoms in the pregenual and subgenual anterior cingulate cortex (ACC), the cingulate cortex, the hippocampus, the parahippocampal gyrus, the superior temporal gyrus, the left cuneus, the right parietal lobe, the posterior insula including the claustrum, the thalamus and the caudate nucleus (Fig. 1A and Supplementary Fig. 7G). We also found brain regions with significant negative correlation (*P* < 0.05, FDRc) between the SUVR_30–50 min_WB of [^11^C]K-2 PET and PANSS score for negative symptoms in the pregenual and subgenual ACC, the left hippocampus, the parahippocampal gyrus, the temporal lobe, the left posterior insula including the claustrum, the thalamus, and the caudate nucleus (Fig. 1B and Supplementary Fig. 7H). Some regions were overlapped between positive and negative symptoms (the pregenual and subgenual ACC, the left posterior part of insula including the claustrum, the left hippocampus, the parahippocampal gyrus, the superior temporal gyrus, the thalamus, and the caudate nucleus), depicting potential core regions for both symptoms of schizophrenia. We also detected symptom-specific areas (positive symptom: the cingulate cortex and the left cuneus, the right parietal lobe, negative symptom: the inferior to middle temporal gyrus). Thus, we identified state brain regions where dysfunction of glutamate synapses can affect the illness severity of schizophrenia.

We performed comparison with SUVR_30–50 min_WB in healthy participants and patients with schizophrenia. Voxel-wise analysis revealed significantly lower SUVR_30–50 min_WB of [^11^C]K-2 PET in patients with schizophrenia in the frontal brain area that consisted of the ACC and the adjacent frontal cortex, and in the lateral brain regions that include the anterior insula and the claustrum, compared to healthy participants (*P* < 0.05, FDRc) (Fig. 1C and Supplementary Fig. 8A). Further, we found a greater SUVR_30–50 min_WB of [^11^C]K-2 PET in the occipital lobe, the putamen, the superior temporal lobe, and the cerebellum of patients with schizophrenia compared to healthy participants (*P* < 0.05, FDRc) of [^11^C]K-2 retention (Fig. 1C and Supplementary Fig. 8A). SUVR_30–50 min_WM of [^11^C]K-2 PET was low in the extensive cortical area, the insula and the cerebellum of patients with schizophrenia, compared to the healthy participants (*P* < 0.05, FDRc) (Supplementary Fig. 8B).

### Characteristics of [^11^C]K-2 in bipolar disorder

Next, we imaged 37 patients with bipolar disorder using [^11^C]K-2. Based on the tTACs (Supplementary Fig. 9A), LGA using the white matter as a reference showed linearity, indicating the reversible binding kinetics of [^11^C]K-2 in patients with bipolar disorder (Supplementary Fig. 9B). As was observed in patients with schizophrenia, there exists a good linear relationship between *BP*_ND_ and SUVR_30–50 min_WM-1 in patients with bipolar disorder (Supplementary Fig. 9C), indicating that SUVR_30–50 min_WM-1 is almost identical to *BP*_ND_. To investigate relative regional alterations in the [^11^C]K-2 PET signal in patients with bipolar disorder, we also constructed the SUVR_30–50 min_WB. There exists a good linear relationship between *BP*_ND_ and SUVR_30–50 min_WB in patients with bipolar disorder (Supplementary Fig. 9D and Supplementary Table 9). The CV among patients with bipolar disorder were lower with SUVR_30–50 min_WB than SUVR_30–50 min_WM, indicating that SUVR_30–50 min_WB has less inter-individual variability. (Supplementary Fig. 9E, F and Supplementary Table 7).

**Fig. 2:**
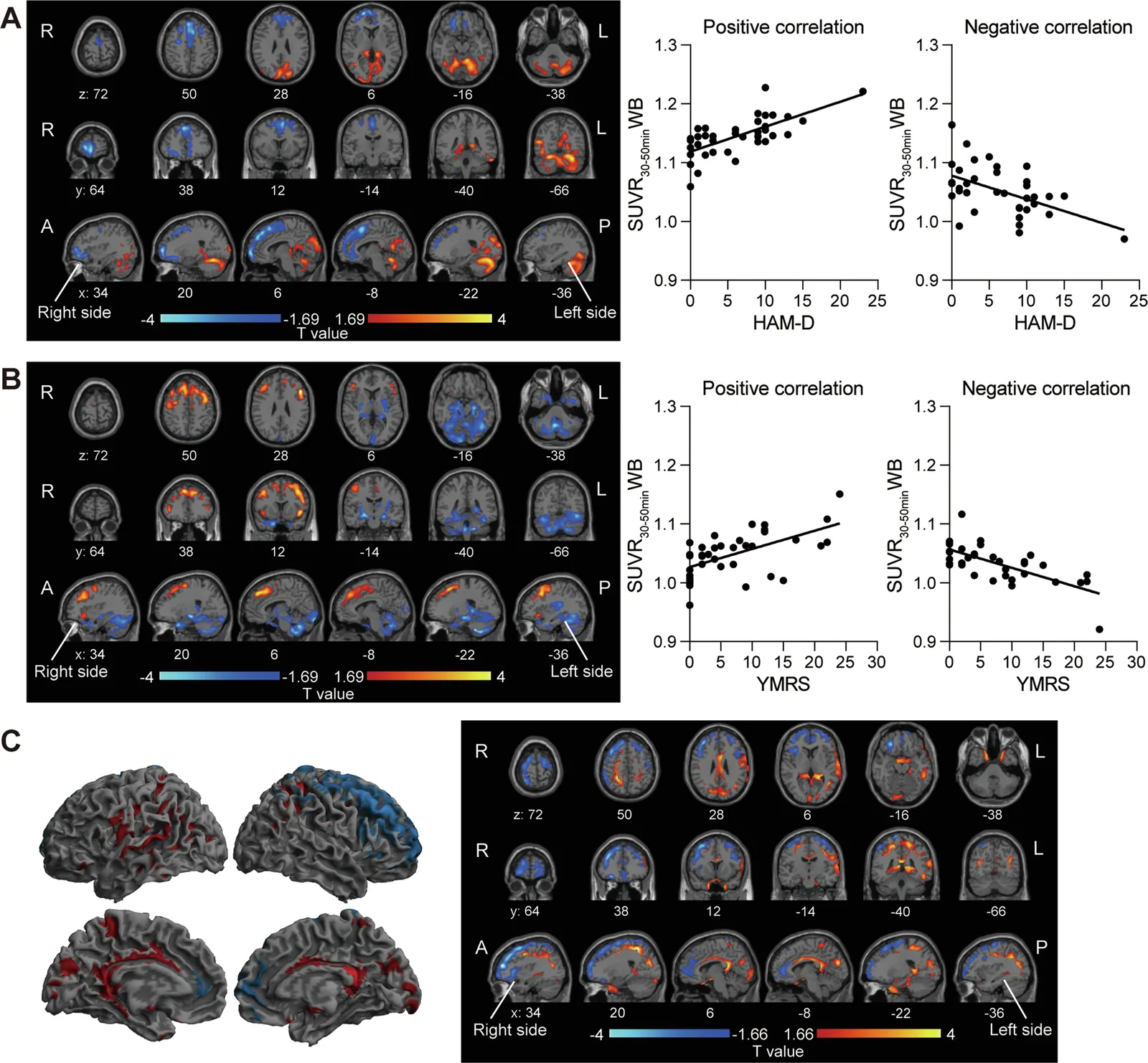
State regions and altered AMPAR distribution in patients with bipolar disorder compared with healthy participants. **A** Brain regions showing a significant negative (blue) and positive (red) correlation between SUVR_30-50min_WB and the 17-item HAM-D score in patients with bipolar disorder (*P* < 0.05, positive correlation: T > 1.69, negative correlation: T < −1.69, one-tailed, FDRc). Significant clusters displayed on an axial, coronal and sagittal slices (Left), and scatter plot between averaged SUVR_30–50 min_WB in significant clusters and HAM-D score (Right, two-tailed Pearson correlation analysis: positive correlation; correlation coefficient = 0.6841, *P* < 0.0001, negative correlation; correlation coefficient = −0.5208, *P* = 0.001). **B** Brain regions showing a significant negative (blue) and positive (red) correlation between SUVR_30–50 min_WB and YMRS score in patients with bipolar disorder (*P* < 0.05, positive correlation: T > 1.69, negative correlation: T < −1.69, one-tailed, FDRc). Significant clusters displayed on an axial, coronal, and sagittal slices (Left), and scatter plot between averaged SUVR_30–50 min_WB in significant clusters and YMRS score (Right, two-tailed Pearson correlation analysis: positive correlation; correlation coefficient = 0.5842, *P* = 0.0001, negative correlation; correlation coefficient = −0.7012, *P* < 0.0001). **C** Relative reduction (blue) and increase (red) of [^11^C]K-2 retention in patients with bipolar disorder compared to healthy participants (*P* < 0.05, increase of [^11^C]K-2 retention: T > 1.66, reduction of [^11^C]K-2 retention: T < −1.66, one-tailed, FDRc). Significant clusters displayed on a 3D-rendered brain (binary image, Left), and axial, coronal, and sagittal slices (T-map, Right). **A**, **B** and **C** (right panel) were adjusted for covariates (age, sex). **C** (left panel) was not adjusted for covariates.

We first investigated the correlation between SUVR_30–50 min_WM and symptomatology scores for bipolar disorders (depressive status; the 17-item HAM-D, manic status; YMRS). Voxel-wise analysis did not reveal brain regions which exhibited correlation between them with statistical significance. As in analysis of schizophrenia, we used SUVR_30–50 min_WB for the analysis. Voxel-wise analysis with SPM exhibited a significant strong negative correlation (*P* < 0.05, FDRc) between the SUVR_30–50 min_WB of [^11^C]K-2 PET (*i.e*., AMPAR density) and the 17-item HAM-D score in the frontal lobe, while we observed a significant positive correlation between these two in the cerebellum and the part of occipital lobe such as the cuneus (Fig. 2A and Supplementary Fig. 9G). In contrast, we detected a significant positive correlation (*P* < 0.05, FDRc) between the AMPAR density and YMRS score in the frontal lobe, while there was a significant negative correlation between them in the cerebellum and a part of occipital lobe such as the cuneus (Fig. 2B and Supplementary Fig. 9H). These results demonstrated that relative alteration of glutamatergic synaptic function of brain regions such as the frontal lobe, cerebellum, and the part of the occipital lobe among all brain regions could be related to control illness state of bipolar disorder. Large brain areas with significant strong correlation between the AMPAR density and HAM-D score were overlapped with those for YMRS score, while some areas were distinct between HAM-D and YMRS, suggesting the existence of core regions producing depressive and manic states and areas specifically for the expression of either depressive or manic symptom (Supplementary Fig. 9I).

AMPAR density characterized with SUVR_30–50 min_WB was lower in the patients with bipolar disorder in the frontal lobe, the right anterior insula, and the anterior cingulate cortex, compared to the healthy participants (*p* < 0.05, FDRc) (Fig. 2C and Supplementary Fig. 10A). We also detected a greater AMPAR density in the occipital lobe and the parietal lobe of patients with bipolar disorder compared to healthy participants (*p* < 0.05, FDRc) (Fig. 2C and Supplementary Fig. 10A). SUVR_30–50 min_WM was decreased in the frontal, parietal and temporal lobe, the anterior insula, and the anterior cingulate cortex of patients with bipolar disorder, compared to the healthy participants (*p* < 0.05, FDRc) (Supplementary Fig. 10B).

### Characteristics of [^11^C]K-2 in depression

We imaged 35 patients with depression using [^11^C]K-2. Based on the tTACs (Supplementary Fig. 11A), LGA using the white matter as a reference showed linearity, indicating the reversible binding kinetics of [^11^C]K-2 (Supplementary Fig. 11B). As was observed in patients with schizophrenia and bipolar disorder, there exists a good linear relationship between *BP*_ND_ and SUVR_30–50 min_WM-1 in patients with depression (Supplementary Fig. 11C), indicating that SUVR_30–50 min_WM-1 is almost identical to *BP*_ND_. To investigate relative regional alterations in the [^11^C]K-2 PET signal in patients with depression, we also constructed the SUVR_30–50 min_WB. There exists a good linear relationship between *BP*_ND_ and SUVR_30–50 min_WB in patients with depression (Supplementary Fig. 11D and Supplementary Table 10). The CV among patients with depression were lower with SUVR_30–50 min_WB than SUVR_30–50 min_WM, indicating that SUVR_30–50 min_WB has less inter-individual variability. (Supplementary Figs. 11E, F and Supplementary Table 7).

**Fig. 3:**
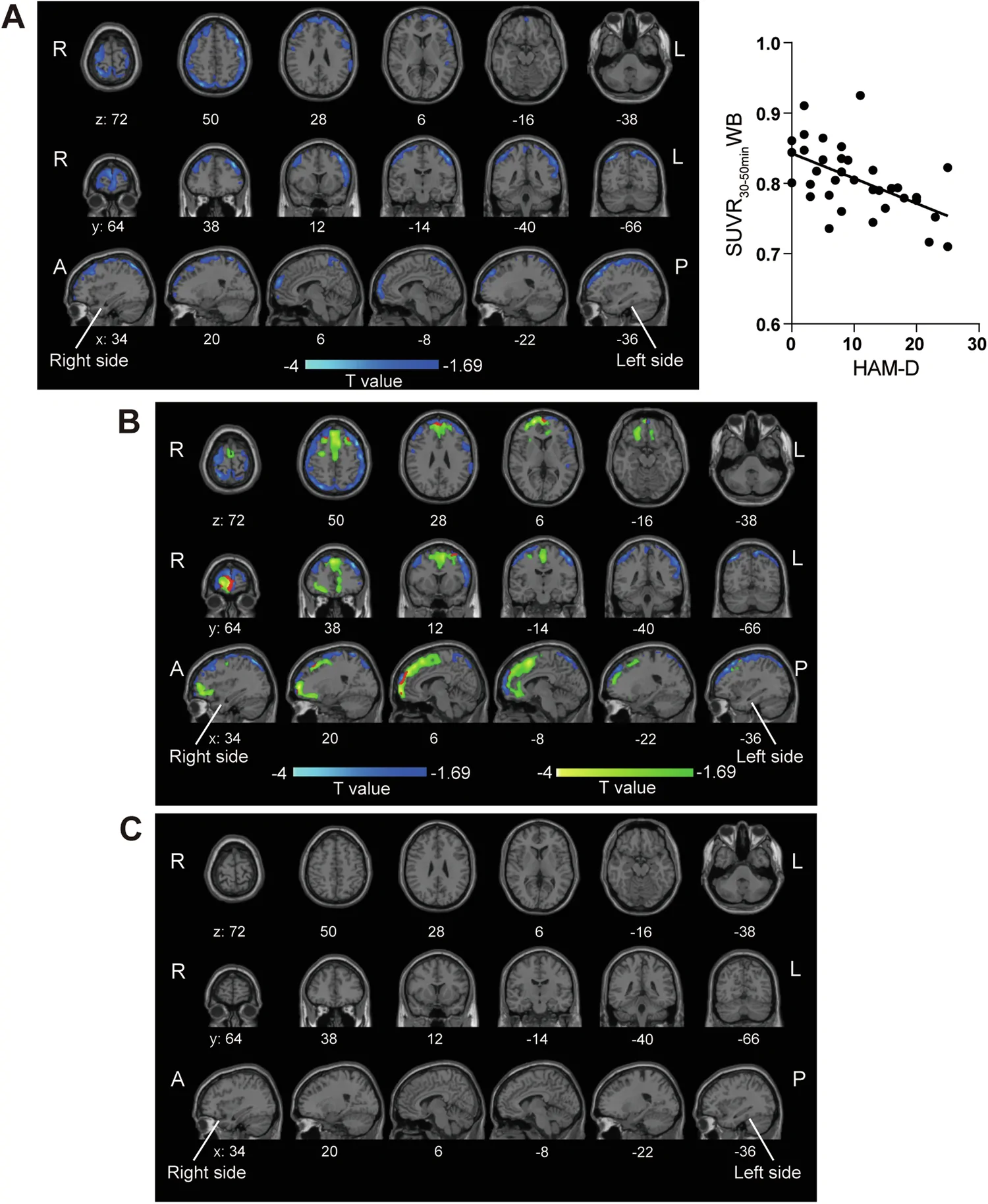
State regions in depression. **A** Brain regions showing a significant negative correlation between SUVR_30–50 min_WB and the 17-item HAM-D score in patients with depression (*P* < 0.05, T < −1.69, one-tailed, FDRc). Significant clusters displayed on an axial, coronal and sagittal slices (Left), and scatter plot between averaged SUVR_30–50 min_WB in significant clusters and HAM-D score (Right, two-tailed Pearson correlation analysis: correlation coefficient = −0.5479, *P* = 0.0007). **B** Brain regions showing a significant negative correlation between SUVR_30–50 min_WB and the 17-item HAM-D score in patients with depression as determined above (blue) and bipolar disorder (green) (*P* < 0.05, T < −1.69, one-tailed, FDRc). Red regions show where the two regions overlap. MNI coordinates for the z-axis (axial slices), y-axis (coronal slices) and x-axis (sagittal slices) were shown bottom of each slice. **C** No significant difference of SUVR_30–50 min_WB between the patients with depression and healthy participants. All panels were adjusted for covariates (age, sex).

We first investigated the correlation between SUVR_30–50 min_WM and symptomatology scores for depression (the 17-item HAM-D). Voxel-wise analysis did not reveal brain regions which exhibited correlation between them with statistical significance. As in analysis of other psychiatric disorders, we used SUVR_30–50 min_WB for the analysis. Voxel-wise analysis with SPM of the patients with depression exhibited a significant negative correlation (*P* < 0.05, FDRc) between the SUVR_30–50 min_WB of [^11^C]K-2 PET and the 17-item HAM-D score in the frontal and parietal lobe (Fig. 3A and Supplementary Fig. 11G). This demonstrated that brain regions such as the frontal lobe and the parietal lobe can be state regions of depression and the unbalanced synaptic functions among these areas underlie depression. Interestingly, these regions were largely different from the brain areas where we detected a significant negative correlation between the SUVR_30–50 min_ of [^11^C]K-2 PET and the 17-item HAM-D score in bipolar disorder (Fig. 3B).

Interestingly, we detected no significant difference of SUVR_30–50 min_WM and SUVR_30–50 min_WB in the brain between the patients with depression and healthy participants (Fig. 3C and Supplementary Fig. 12).

### Characteristics of [^11^C]K-2 in ASD

We next profiled AMPAR in 35 patients with ASD using [^11^C]K-2. Based on the tTACs (Supplementary Fig. 13A), LGA using the white matter as a reference exhibited linearity, suggesting the reversible binding kinetics of [^11^C]K-2 (Supplementary Fig. 13B). As was observed in patients with other psychiatric disorders, there exists a good linear relationship between *BP*_ND_ and SUVR_30–50 min_WM-1 in patients with ASD (Supplementary Fig. 13C), indicating that SUVR_30–50 min_WM-1 is almost identical to *BP*_ND_. To investigate relative regional alterations in the [^11^C]K-2 PET signal in patients with ASD, we also constructed the SUVR_30–50 min_WB. There exists a good linear relationship between *BP*_ND_ and SUVR_30–50 min_WB in patients with ASD (Supplementary Fig. 13D and Supplementary Table 11). The CV among patients with ASD were lower with SUVR_30–50 min_WB than SUVR_30–50 min_WM, indicating that SUVR_30–50 min_WB has less inter-individual variability. (Supplementary Fig. 13E, F and Supplementary Table 7).

**Fig. 4:**
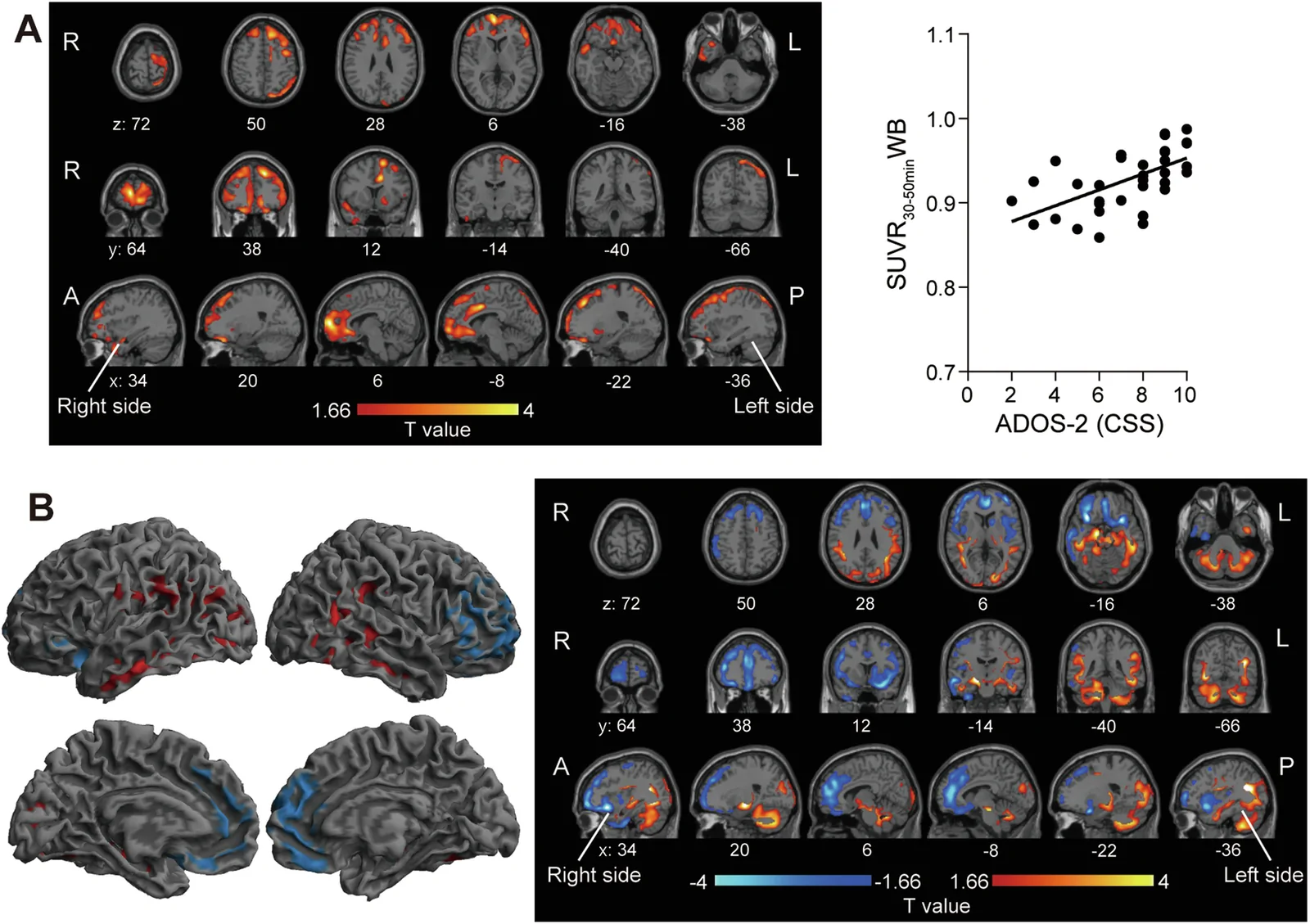
State regions and altered AMPAR distribution in patients with ASD compared with healthy participants. **A** Brain regions showing a significant positive correlation between SUVR_30–50 min_WB and ADOS-2 Module 4 CSS in patients with ASD (*P* < 0.05, T > 1.69, one-tailed, FDRc). Significant clusters displayed on an axial, coronal, and sagittal slices (Left), and scatter plot between averaged SUVR_30–50 min_WB in significant clusters and ADOS-2 Module 4 CSS (Right, two-tailed Pearson correlation analysis: correlation coefficient = 0.6027, *P* = 0.0001). **B** Relative reduction (blue) and increase (red) of [^11^C]K-2 retention in patients with ASD compared to healthy participants (*P* < 0.05, increase of [^11^C]K-2 retention: T > 1.66, reduction of [^11^C]K-2 retention: T < −1.66, one-tailed, FDRc). Significant clusters displayed on a 3D-rendered brain (binary image, Left), and axial, coronal and sagittal slices (T-map, Right). **A** and **B** (right panel) were adjusted for covariates (age, sex). **B** (left panel) was not adjusted for covariates.

We first investigated the correlation between SUVR_30–50 min_WM and symptomatology scores for ASD (ADOS-2 Module 4 CSS). Voxel-wise analysis did not reveal brain regions which exhibited correlation between them with statistical significance. As in analysis of other psychiatric disorders, we used SUVR_30–50 min_WB for the analysis. We found a significant positive correlation (*P* < 0.05, FDRc) between the SUVR_30–50 min_WB of [^11^C]K-2 PET and the ADOS-2 Module 4 CSS in the large cortical areas such as the orbitofrontal cortex, the ACC, and the frontal and parietal lobe, indicating that these cortical areas are state brain regions for ASD (Fig. 4A and Supplementary Fig. 13G).

SUVR_30–50 min_WB of [^11^C]K-2 PET was lower in the patients with ASD in the right middle frontal gyrus, the anterior cingulate gyrus, and the left anterior insula (*P* < 0.05, FDRc) (Fig. 4B and Supplementary Fig. 14A). We also detected a greater SUVR_30–50 min_ of [^11^C]K-2 in the occipital lobe, the inferior temporal lobe, and the cerebellum of patients with ASD compared to healthy participants (*P* < 0.05, FDRc) (Fig. 4B and Supplementary Fig. 14A). SUVR_30–50 min_WM was decreased in the frontal, parietal and temporal lobe, the anterior insula, and the anterior cingulate cortex of patients with ASD, compared to the healthy participants (*p* < 0.05, FDRc) (Supplementary Fig. 14B).

### Identification of commonly affected areas in terms of AMPAR distribution across psychiatric disorders

**Fig. 5:**
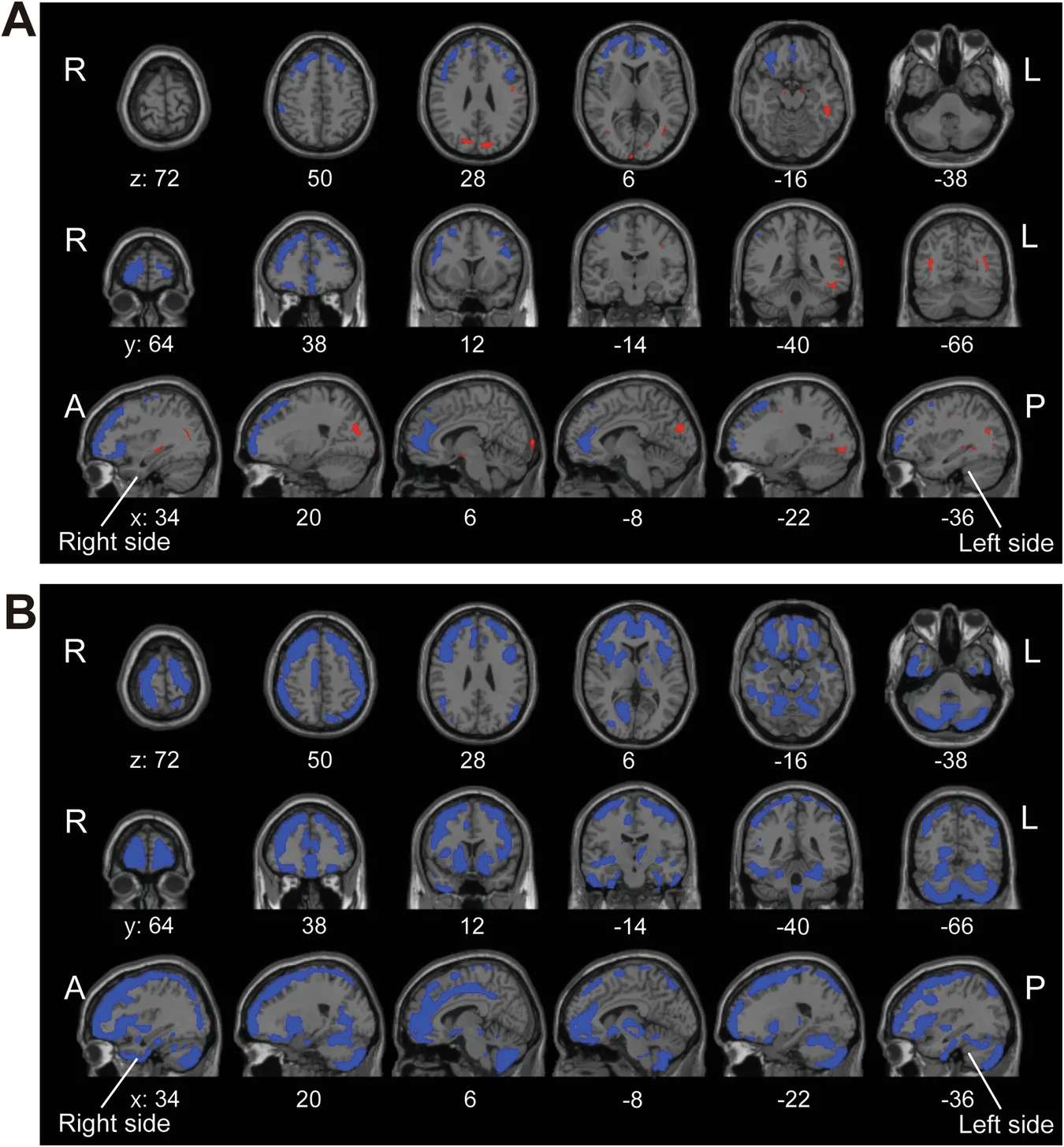
Commonly affected areas across psychiatric disorders. **A** Common brain regions showing reduced (blue) and increased (red) SUVR_30–50 min_WB, respectively, in patients with schizophrenia, bipolar disorder and ASD compared with healthy participants. **B**　Common brain regions showing reduced (blue) SUVR_30–50 min_WM, in patients with schizophrenia, bipolar disorder and ASD compared with healthy participants. MNI coordinates for the z-axis (axial slices), y-axis (coronal slices) and x-axis (sagittal slices) were shown bottom of each slice.

Data of the participants with schizophrenia, bipolar disorder, and ASD that possess brain regions with altered AMPAR distribution compared to healthy participants were used for this analysis. We found that SUVR_30–50 min_WB was commonly decreased in the ACC, superior frontal gyrus, middle frontal gyrus, the orbital gyrus, the rectus, and the anterior part of insula and increased in the superior temporal gyrus, and the cuneus compared with healthy participants across these disorders (Fig. 5A and Supplementary Fig. 15A). We also found that SUVR_30–50 min_WM was commonly decreased in the anterior to middle cingulate gyrus, the frontal gyrus, the orbital gyrus, the rectus, the parietal gyrus, the middle occipital gyrus, the right lingual gyrus, the anterior part of insula, the putamen, the caudate, the pallidum, the left thalamus, and the cerebellum (Fig. 5B and Supplementary Fig. 15B).

## Discussion

The present study has revealed that unbalanced glutamatergic synaptic functions, assessed with AMPAR density, among brain regions can affect distinct and diverse status of psychiatric disorders. While we did not detect brain regions which exhibited significant correlation between SUVR_30–50 min_WM and symptomatology scores of diseases investigated, we found brain regions with significant correlations between SUVR_30–50 min_WB and symptomatology scores in these diseases. As was presented in Supplementary Figs. 6C, E, 7E, F, 9E, F, 11E, F, 13E, F and Supplementary Table 7, the CV among individuals were relatively larger in SUVR_30–50 min_WM than SUVR_30–50 min_WB. While SUVR_30–50 min_WM reflects absolute AMPAR density in the brain, SUVR_30–50 min_WB represents the balance of regional AMPAR density among brain regions. Further, since psychiatric disorders are considered to be the disruption of networks among brain regions whose fundamentals are synaptic function. Therefore, we could detect significant correlations between SUVR_30–50 min_WB and symptomatology scores but not SUVR_30–50 min_WM and, thus, SUVR_30–50 min_WB can be the optimal correction to analyze psychiatric disorders as “synapse diseases” in the network. SUVR_30–50 min_WM exhibited the reduction of AMPAR density in large brain areas especially in schizophrenia and ASD than healthy participants (Supplementary Figs. 8B and 14B), while SUVR_30–50 min_WB depicted region specific reduction or increase of AMPAR density in schizophrenia, bipolar disorder, and ASD (Figs. 1C, 2C, 4B and Supplementary Figs. 8A, 10A and 14A). Thus, systemic reduction and unbalanced distribution among brain regions of AMPAR can underlie the pathogenesis of psychiatric disorders.

We observed interesting synaptic features in areas which exhibited significant correlation between symptomatology scores and AMPAR density. In schizophrenia, while some brain areas for positive symptoms were overlapped with those for negative symptoms, there were specific areas either for positive or negative symptoms. The existence of overlapped and non-overlapped areas for these symptoms implies that the common circuit and symptom-specific circuits regulate the expression of positive and negative symptoms (Fig. 1A, B and Supplementary Fig. 7G, H). In bipolar disorder, the balance of synaptic functions between the frontal lobe and occipital lobe/cerebellum can control moods such as depressive and manic state (Fig. 2A, B and Supplementary Fig. 9G, H). Since some regions were overlapped between those correlated with depressive and manic state (Supplementary Fig. 9I), depressive and manic states in bipolar disorder might be biologically related. In depression, we detected a negative correlation between HAM-D score and SUVR_30–50 min_ with [^11^C]K-2 (Fig. 3A and Supplementary Fig. 11G). While this phenotype in the frontal lobe of depression is similar to that of bipolar disorder, brain regions with this correlation in the frontal lobe were not largely overlapped between these disorders (Fig. 3B). Biological characterization of this observation warrants further investigations. In ASD, we detected a positive correlation between ADOS-2 severity score and SUVR_30–50 min_ with [^11^C]K-2 in large part of the cortex (Fig. 4A and Supplementary Fig. 13G). This suggests that elevated synaptic activity can disrupt the signal-to-noise ratio during the processing of information such as sensory perception, resulting in the overflow of external inputs which is often observed in patients with ASD [36].

We also detected brain regions exhibiting altered AMPAR density in patients with schizophrenia, bipolar disorder, and ASD, compared to healthy controls; moreover, some regions such as anterior cingulate gyrus, insula, and cuneus were overlapped across these three disorders. Biological characterization of these regions remains to be elucidated. An interesting observation is that we did not detect brain regions with a difference of the average SUVR_30–50 min_ using [^11^C]K-2 between patients with depression and healthy controls. This finding suggests that there is a continuum between depression and normal condition, unlike schizophrenia, bipolar disorder, and ASD. Our approach to elucidate glutamatergic synaptic functions, using the novel PET tracer, beyond the border of conventional diagnoses can be interpreted in the context of Research Domain Criteria (RDoC) [37]. This line of research will in turn enable the development of a novel diagnostic system coupled with therapeutics based on the biological profiling of psychiatric patients in terms of AMPAR, such as AMPAR blockers for the condition with increased AMPAR density and brain stimulation targeting a specific area that shows decreased AMPAR density.

In schizophrenia, bipolar disorder, and ASD, there was almost no overlap between the regions where clinical scores correlated with AMPAR density (state regions) and those where there were differences in AMPAR density compared to healthy participants. In our hypothesis, the regions with differences compared to healthy participants (but there is no correlation between illness severity and AMPAR density) may exist as ‘trait’ regions of the disease. “Trait” regions can exist regardless of illness severity (can exist even before the onset of the disease). On the other hand, the state regions are brain areas where AMPAR density can directly affect illness severity. We hypothesize that the trait region is formed first potentially due to the genetic factors etc., and then some kind of stress can induce changes in AMPAR in the state region and produces symptoms. It is interesting to know the order of formation of trait regions (ACC, insula, and occipital lobe) and how they generate the state region. However, we cannot rule out the possibility that these regions are secondary effects or not, and this is the limitation of this study. Our hypothesis can be proven by studies with animal models and longitudinal studies in human.

This study is a cross-sectional observation, limiting the causality between the AMPAR expression and the disease entity and behavior. One strength of our approach to psychiatric disorders in human based on the AMPAR is that we can further characterize causal relationship between phenotypes in the distribution of AMPAR and pathogenesis using animal experiments in which we can easily manipulate the expression of AMPAR in specific bran regions. We need further characterization of the above-mentioned brain regions using animal experiments for elucidation of the causal relationship between AMPAR phenotype observed in patients and pathogenesis, circuit dissection responsible for these disorders, and understanding of how commonly affected areas across diseases are generated and regions associated with each psychiatric symptom are created. Our approach to psychiatric disorders using [^11^C]K-2 can elucidate the biological mechanisms across diseases and pave the way to develop novel diagnostics and therapeutics based on the synapse physiology.

## Supplementary information

Supplementary information

## Data Availability

All requests for raw and analyzed data are promptly reviewed by the Yokohama City University Research Promotion Department to determine whether the request is subject to any intellectual property or confidentiality obligations and, further, inspected by the Institutional Review Board of Yokohama City University Hospital. Upon these approvals, derived data will be released via a material transfer agreement from the corresponding author.
