# Supplementary material for: Characterization of patients with major psychiatric disorders with AMPA receptor positron emission tomography
Source: Mol Psychiatry. 2024 Oct 15;30(5):1780–90. doi: 10.1038/s41380-024-02785-1 (PMC12014498; doi:10.1038/s41380-024-02785-1)
Supplement: Supplementary file 1 — Supplementary information [file 41380_2024_2785_MOESM1_ESM.docx]

**Supplementary information**

**Title:** **Characterization of patients with major psychiatric disorders with AMPA receptor positron emission tomography**

**Authors: Authors:** Mai Hatano^1#^, Waki Nakajima^1#^, Hideaki Tani^2#^, Hiroyuki Uchida^2^, Tomoyuki Miyazaki^1,3^, Tetsu Arisawa^4^, Yuuki Takada^1^, Sakiko Tsugawa^1,2^ Akane Sano^1^, Kotaro Nakano^1^, Tsuyoshi Eiro^1^, Hiroki Abe^1^, Akira Suda^5^, Takeshi Asami^5^, Akitoyo Hishimoto^6^,　Nobuhiro Nagai^2^, Teruki Koizumi^2^, Shinichiro Nakajima^2^, Shunya Kurokawa^2^, Yohei Ohtani^2^, Kie Takahashi^2^, Yuhei Kikuchi^2^, Taisuke Yatomi^2^, Shiori Honda^2^, Masahiro Jinzaki^7^, Yoji Hirano^8,9,10^, Ryo Mitoma^9^, Shunsuke Tamura^8,9^, Shingo Baba^11^, Osamu Togao^11^, Hirotaka Kosaka^12^, Hidehiko Okazawa^13^, Yuichi Kimura^14^, Masaru Mimura^2^, and Takuya Takahashi^1,15*^

**Affiliations:**

^1^ Department of Physiology, Yokohama City University Graduate School of Medicine, Yokohama, 236-0004, Japan

^2^ Department of Neuropsychiatry, Keio University School of Medicine, Tokyo, 160-8582, Japan

^3^ Center for Promotion of Research and Industry-Academic Collaboration, Yokohama City University, Yokohama, 236-0004, Japan

^4^ Radioisotope Research Center, Yokohama City University Graduate School of Medicine, Yokohama, 236-0004, Japan

^5^ Department of Psychiatry, Yokohama City University Graduate School of Medicine, Yokohama, 236-0004, Japan

^6^ Department of Psychiatry, Kobe University Graduate School of Medicine, Kobe, 650-0017, Japan

^7^ Department of Radiology, Keio University School of Medicine, Tokyo, 160-8582, Japan

^8^ Department of Psychiatry, Division of Clinical Neuroscience, Faculty of Medicine, University of Miyazaki, Miyazaki, 889-1692, Japan

^9^ Department of Neuropsychiatry, Graduate School of Medical Sciences, Kyusyu University, Fukuoka, 812-8582, Japan

^10^ Institute of Industrial Science, The University of Tokyo, Tokyo, 153-8505, Japan

^11^ Department of Clinical Radiology, Graduate School of Medical Sciences, Kyusyu University, Fukuoka, 812-8582, Japan

^12^ Department of Psychiatry, Faculty of Medical Sciences, University of Fukui, Fukui, 910-1193, Japan

^13^ Biomedical Imaging Research Center, University of Fukui, Fukui, 910-1193, Japan

^14^ Faculty of Informatics, Cyber Informatics Research Institute, Kindai University, Higashi-Osaka, 577-8502, Japan

^15^ The International Research Center for Neurointelligence, Institutes for Advanced Study, University of Tokyo, Tokyo, 113-8654, Japan

#These authors contributed equally to this work.

* To whom correspondence should be addressed

**Supplemental Methods**

**Sample size**

This study comprised five clinical studies. Since the rationale for setting the sample size is different for each study, it is described below for each study.

***Clinical study of multiple diagnosis (pilot study)*** (UMIN000025132): A power-based sample size design based on a two-sample t-test was used. In a previous study[Gibbons e et al], the mean AMPAR density values in anterior cingulate cortex for healthy group and psychiatric disorder group were roughly 210 fmol/mg and 280 fmol/mg, respectively, with a standard deviation of 50. As in the previous study, the standardized difference between groups = difference in means/standard deviation = (280-210)/50 = 1.4, which is estimated to ensure 84% power with 9 subjects in each group at a two-sided significance level of 5%. Based on the above, we set the target number of patients in each group at 10, considering the possibility of a few cases being excluded from the analysis.

***Clinical study of ASD*** (jRCTs031190149): Based on the results of the pilot study, AMPAR density in the right middle frontal gyrus and left superior frontal gyrus correlates with ADOS-2 (CSS) (correlation coefficient >0.7). In this study, the correlation coefficient was conservatively estimated to be 0.6 to calculate the number of cases. Assuming the correlation coefficient of 0.6 or higher between AMPAR density and severity, a two-sided significance level of 2.5% and a power of 90% would require 29 sets of data. Considering the possibility of a few cases being excluded from the analysis, we set the target number of cases at 30.

***Clinical study of depression and bipolar disorder*** (jRCTs031190150): Considering result of pilot study, assuming the correlation coefficient of 0.5 or higher between AMPAR density and severity, a two-sided significance level of 5% and a power of 80% would require 29 sets of data in clinical study of depression and bipolar disorder. Considering the possibility of a few cases being excluded from the analysis, we set the target number of cases at 30.

***Clinical study of schizophrenia*** (jRCTs031190197): Based on the results of the pilot study, AMPAR density in the right middle temporal gyrus and right anterior cingulate cortex correlates with PANSS (correlation coefficient >0.6). Assuming the correlation coefficient of 0.6 or higher between AMPAR density and severity, a two-sided significance level of 2.5% and a power of 90% would require 29 sets of data. Considering the possibility of a few cases being excluded from the analysis, we set the target number of cases at 30.

***Clinical study of healthy participants*** (jRCTs031200083): This study aimed to improve the accuracy of the correlation between age and AMPAR density in healthy participants, and considering the feasibility of recruiting healthy participants, a target number of 10 patients per age group (20s, 30s, 40s, 50s, and 60s) and per sex (male and female) (100 patients in total) was set.

**In- and exclusion criteria**

**Patients with schizophrenia.** In the first study (UMIN000025132), the inclusion criteria were male in- and outpatients 30–49 years of age who were capable of providing informed consent; and patients who were diagnosed with schizophrenia according to the Diagnostic and Statistical Manual of Mental Disorders Fourth Edition (DSM-IV) ^1^, using the structured clinical interview for DSM-IV (SCID-I/DSM-IV) ^2^, the DSM Fifth Edition (DSM-5) ^3^, and the International Classification of Diseases Tenth Edition (ICD-10) ^4^. In the second study (jRCTs031190197), the inclusion criteria were the same as those in the first study, other than the age range (i.e., 20-59 years) and sex (i.e., both men and women were included). Exclusion criteria were the same for both studies; participants were excluded if they were pregnant, nursing, or desired to become pregnant; had a history of epilepsy; met substance abuse criteria within six months of the study; had a positive urine drug screen for illicit drugs; received treatment with perampanel; met contraindications for MRI scan; had significant neurological or general medical conditions; or showed abnormal laboratory test values of serum creatinine ≥ 1.5 mg/dl, aspartate aminotransferase (AST) ≥ 150 IU/L, or alanine aminotransferase (ALT) ≥ 150 IU/L.

**Patients with bipolar disorder.** In the first study (UMIN000025132) the inclusion criteria were male in- and outpatients 30–49 years of age who were capable of providing informed consent; and patients who were diagnosed with bipolar disorder according to the DSM-IV ^1^, using the SCID-I/DSM-IV ^2^, the DSM-5 ^3^, and the ICD-10 ^4^. In the second study (jRCTs031190150), the inclusion criteria were the same as those in the first study, other than the age range (i.e., 20-59 years) and sex (i.e., both men and women were included). Exclusion criteria were the same for both studies; participants were excluded if they were pregnant, nursing, or desired to become pregnant; had a history of epilepsy; met criteria for substance abuse within six months of the study; had a positive urine drug screen for illicit drugs; received treatment with perampanel; met contraindications for MRI scan; had significant neurological or general medical conditions; or had abnormal laboratory test values of serum creatinine ≥ 1.5 mg/dl, AST ≥ 150 IU/L, or ALT ≥ 150 IU/L.

**Patients with depression.** In the first study (UMIN000025132) the inclusion criteria were: male in- and outpatients 30–49 years of age who were capable of providing informed consent; and patients who were diagnosed with major depressive disorder according to the DSM-IV ^1^, using the SCID-I/DSM-IV ^2^, the DSM-5 ^3^, and the ICD-10 ^4^. In the second study (jRCTs031190150), the inclusion criteria were the same as those in the first study, other than the age range (i.e., 20-59 years) and sex (i.e., both men and women were included). Exclusion criteria were the same for both studies; participants were excluded if they were pregnant, nursing, or desired to become pregnant; had a history of epilepsy; met the substance abuse criteria within six months of the study; had a positive urine drug screen for illicit drugs; received treatment with perampanel; met contraindications for MRI scan; had significant neurological or general medical conditions; or had abnormal laboratory test values of serum creatinine ≥ 1.5 mg/dl, AST ≥ 150 IU/L, or ALT ≥ 150 IU/L.

**Patients with autism spectrum disorder.** In the first study (UMIN000025132) the inclusion criteria were male in- and outpatients 30–49 years of age who were capable of providing informed consent; and patients who were diagnosed with autism spectrum disorder according to the DSM-5 ^3^. In the second study (jRCTs031190149), the inclusion criteria were the same as those in the first study, other than the age range (i.e., 20-59 years), sex (i.e., both men and women were included), and intellectual performance (i.e., full scale intelligence quotient [FIQ] ≥ 70 according to Wechsler Adult Intelligence Scale third edition [WAIS-III] or Wechsler Adult Intelligence Scale fourth edition [WAIS-IV]). Exclusion criteria were the same for both studies; participants were excluded if they were pregnant, nursing, or desired to become pregnant; had a history of epilepsy; met the substance abuse criteria within six months of the study; had a positive urine drug screen for illicit drugs; received treatment with perampanel; met contraindications for MRI scan; had significant neurological or general medical conditions; or had abnormal laboratory test values of serum creatinine ≥ 1.5 mg/dl, AST ≥ 150 IU/L, or ALT ≥ 150 IU/L.

**Healthy participants.** In the first study (UMIN000025132) the inclusion criteria were healthy male participants who were 30-79 years of age who were capable of providing informed consent and did not fulfil any diagnostic criteria for psychiatric conditions according to the DSM-IV ^1^ using the SCID-I/DSM-IV ^2^, DSM-5 ^3^ or ICD-10 ^4^. Among them, age-matched (i.e., 30-59 years) healthy participants were included. In the second study (jRCTs031200083), the selection criteria were the same as those in the first study, other than the age range (i.e., 20-49 years) and sex (i.e., both men and women were included). Exclusion criteria were the same for the two studies; participants were excluded if they were pregnant, nursing, or desired to become pregnant; had a history of epilepsy, met criteria for substance abuse within six months of the study; had a positive urine drug screen for illicit drugs; received treatment with perampanel; met contraindications for MRI scan; had significant neurological or general medical conditions; or had abnormal laboratory test values of serum creatinine ≥ 1.5 mg/dl, AST ≥ 150 IU/L, or ALT ≥ 150 IU/L.

**Settings and procedures of positron emission tomography (PET) and magnetic resonance imaging (MRI)**

**Settings.** The participants underwent a PET scan with [^11^C]K-2 and an MRI scan. [^11^C]K-2 was synthesized locally at each site in accordance with GMP ordinance. Injected dose of [^11^C]K-2 was 374.3 ± 11.9 MBq (healthy participants), 381.5 ± 11.1 MBq (patients with schizophrenia), 376.2 ±11.7 MBq (patients with bipolar disorder), 368.7 ± 14.0 MBq (patients with depression) and 374.0 ± 13.2 MBq (patients with ASD).

*Yokohama City University Hospital.* PET imaging was performed with a TOSHIBA Aquiduo scanner (TOSHIBA Medical) and Celesteion PCA-9000A/2A (Canon medical). Aquiduo provided an axial FOV of 240 mm, and 80 contiguous 2.0 mm thick slices. A 4.7 s transmission scan was performed for attenuation correction (AC), then a 60 s intravenous injection of [^11^C]K-2 was given, which was followed by an emission scan of 60 min in all studies, with frames of 18 × 10 s, 2 × 30 s, 7 × 60 s, 1 × 2 min, 1 × 3 min, 3 × 5 min and 3 × 10 min. Dynamic images were reconstructed with a 2D-OSEM using 4 iterations, 14 subsets, a 128 matrix, a zoom of 2.8 and a 5.0 mm Gaussian filter. Celesteion provided an axial FOV of 240 mm and 96 contiguous 2.0 mm-thick slices. A 15.2 s transmission scan was performed for AC, and a 60 s intravenous injection of [^11^C]K-2 was administered, followed by an emission scan of 60 min, with 35 frames. Dynamic images were reconstructed with 3D-OSEM + TOF using 2 iterations, 20 subsets, a 128 matrix, a zoom of 1.0, and a 5.0-mm Gaussian filter. To permit accurate delineation of the brain regions for data analysis, each participant underwent an MRI scan on a GE DISCOVERY MR750 (General Electric Medical Systems). High resolution 3D-T1-weighted images (T1WI) were acquired using the following parameters: voxel size = 0.9 × 0.9 × 0.9 mm, repetition time (TR) / time to echo (TE) = 7.0/3.1 ms, flip angle (FA) = 8°, FOV = 220 mm, Matrix = 256 × 256.

*Keio University Hospital.* PET imaging was performed with a Biograph mCT Flow (Siemens Medical Solutions), which provided an axial FOV of 300 mm, and 111 contiguous 2.0 mm thick slices. A 6.56 s transmission scan was performed for AC, then a 60 s intravenous injection of [^11^C]K-2 was given, which was followed by an emission scan of 60 min, with 35 frames. Dynamic images were reconstructed with a 3D-OSEM + TOF using 4 iterations, 24 subsets, a 200 matrix, a zoom of 2.0 and a 2.0 mm Gaussian filter. To permit accurate delineation of the brain regions for data analysis, each participant underwent an MRI scan on a MAGNETOM Prisma (SIEMENS Healthineers) at university of Tokyo. High resolution 3D-T1WI were acquired using 3D MPRAGE protocol (voxel size = 0.8 × 0.8 × 0.8 mm, TR/TE = 2400/2.22 ms, FA = 8°, FOV = 208 mm, Matrix = 300 × 320).

*Kyushu University Hospital.* PET imaging was performed with a Biograph mCT Flow (Siemens Medical Solutions) and a Biograph Vision (Siemens Medical Solutions). Biograph mCT provided an axial FOV of 250 mm, and 165 contiguous 1.0 mm thick slices. A 9.38 s transmission scan was performed for AC, then a 60 s intravenous injection of [^11^C]K-2 was given, which was followed by an emission scan of 60 min, with 35 frames. Dynamic images were reconstructed with a 3D-OSEM + TOF using 5 iterations, 21 subsets and a 5.0 mm Gaussian filter. Biograph Vision provided an axial FOV of 357 mm, and 263 contiguous 1.0 mm thick slices. A 26.67 s transmission scan was performed for AC, then a 60 s intravenous injection of [^11^C]K-2 was given, which was followed by an emission scan of 60 min, with 35 frames. Dynamic images were reconstructed with a 3D-OSEM + TOF using 8 iterations, 5 subsets and a 5.0 mm Gaussian filter. To permit accurate delineation of the brain regions for data analysis, each participant underwent an MRI scan on an Ingenia 3.0-T scanner (Phillips). High resolution 3D-T1WI were acquired using 3D MPRAGE protocol (voxel size = 1.2 × 1.0 × 1.0 mm, TR/TE = 6.8/3.1 ms, FA = 9°, FOV = 170 mm, Matrix = 256 × 256).

*University of Fukui Hospital.* PET and MRI were simultaneously scanned with a Signa PET/MRI (GE Healthcare). Signa PET/MRI provided an axial FOV of 256 mm, and 89 contiguous 2.78 mm thick slices. For AC, a 3D radial MR acquisition for the zero-echo time (ZTE) method in the axial direction was performed with the following sequence: FOV 264 mm, matrix 110 × 110 × 116, voxel size 2.4 × 2.4 × 2.4 mm^3^, flip angle 0.8°, number of excitations 4, bandwidth ± 62.5 kHz and acquisition time of 41 s.　The ZTE-AC method was previously described.^5^ A 60 s intravenous injection of [^11^C] K-2 was performed and followed by an emission scan of 60 min, with 35 frames. Dynamic images were reconstructed with a 3D-OSEM + TOF using 3 iterations, 28 subsets, a 128 matrix, and a 3.0 mm Gaussian filter. High resolution 3D-T1WI and other MR images were acquired simultaneously using the following sequence: voxel size = 0.9 × 0.9 × 0.9 mm, TR/TE = 8.5/3.3 ms, FA = 8°, FOV = 196 mm, Matrix = 256 × 256).

**Acquisition of tissue time activity curve.** To match PET image resolution, dynamic PET images were processed with a 5 mm FWHM Gaussian filter for the participants scanned at Keio University Hospital and the University of Fukui hospital. For each participant, PET images and T1WI were spatially normalized into Montreal Neurological Institute (MNI) standard space with the PMOD PNEURO tool version 3.709 (PMOD Technologies). Region of Interests (ROIs) were automatically obtained from brain areas encoded in the N30R83 maximum-probability atlas.^6^ For the VOI of white matter (WM), it was obtained by fulfilling the following conditions for voxel value: the probability of the presence of the WM >0.9, the 8 mm-smoothed gray matter (GM) <0.05 and the 8 mm-smoothed cerebrospinal fluid (CSF) <0.05 with Statistical Parametric Mapping (SPM) 8.^7^ Tissue time activity curves (tTACs) were generated using ROIs of these brain regions. Standardized uptake value (SUV) for each brain regions were calculated as follows: radioactivity concentration in the region at time *t* (Bq ml^-1^)/ (injected dose (Bq)/body weight (kg)).

**PET and MRI analysis procedure.** A summed PET image at 30-50 min after injection of [^11^C]K-2 was obtained for each patient. SUV ratio (SUVR)_30-50 min_ images were normalized by white matter mean value (SUVR_30-50min_WM) or whole brain mean value (SUVR_30-50min_WB). We created the template for normalization from T1WIs of all participants using the high-dimensional nonlinear warping algorithm DARTEL ^8^. SUVR_30-50 min_ images were spatially normalized into MNI standard space using the template with SPM 12. In more detail, the SUVR_30-50 min_ image and T1WI were firstly co-registered, then the T1WI were segmented into probability maps of GM, WM, and CSF using a unified framework for tissue segmentation ^9^. For the participants scanned at Keio University Hospital, T1-weighted images co-registered to T2-weighted images using advanced normalization tools ^10-12^, due to artifacts of the bottom of frontal lobe. Then, multi-spectral segmentation was performed to T1- and T2-weighted images. The DARTEL algorithm was applied to the segmented GM and WM images to calculate the spatial transformation required to normalize each individual’s images, resulting in creating the study population template and each flow field image having a form of each participant’s brain. Normalization from native image space to MNI standard space was performed to individual SUVR_30-50min_ images co-registered by their T1-weighted MRI images using the study population template and their flow field images. PET images were normalized by preserving concentration. The spatially normalized images were finally processed with an 8 mm FWHM Gaussian filter.

**PET camera validation using phantom.** To determine reconstruction parameters of PET images, and validate PET cameras, we performed PET scan on all types of PET cameras using brain tumor (BT) phantom (Itoi Factory Inc.) ^13^, which has multiple spheres of different sizes (diameter 7.5, 10, 13, 16, 27, 38mm) placed inside the cavity. The background area and the spheres of the BT phantom were filled with the activity of 5 and 10 kBq/ml using ^18^F-FDG, respectively. The evaluation was made based on three criteria: 1) recovery rate, 2) uniformity, and 3) quantitative performance. How to set up a circular ROI for recovery analysis: Perform this on the slice in which the sphere of each size is most clearly depicted. Set an ROI with the same size as each sphere and 10 circular ROIs with a size of about 100 mm^2^ in the background area (6 pieces at a distance of 15 mm or more from the phantom edge and 4 pieces at the center). How to set ROI for uniformity analysis: Perform this for three slices in which no sphere is depicted. Use slices separated by at least 10 mm from each other. Set 16 circular ROIs with a size of about 100 mm^2^ in the background area. (12 at a distance of 15 mm or more from the phantom edge and 4 at the center) For each ROI, calculate the average value of SUV (SUVmean). The evaluation criteria were defined as follows. 1) recovery rate : SUVmean of each HOT sphere is 90% or more for 38mm sphere, 85% or more for 27mm sphere, 70% or more for 20mm sphere, and 60% or more with 16mm sphere. 2) Uniformity: The standard deviation of the relative error of SUVmean for the set ROI is 0.0249 or less. (C) quantitative performance: the SUVmean of the set ROI should fall within the range of 0.95 to 1.05 against the theoretical value. We confirmed that all PET cameras used in this study could meet these criteria.


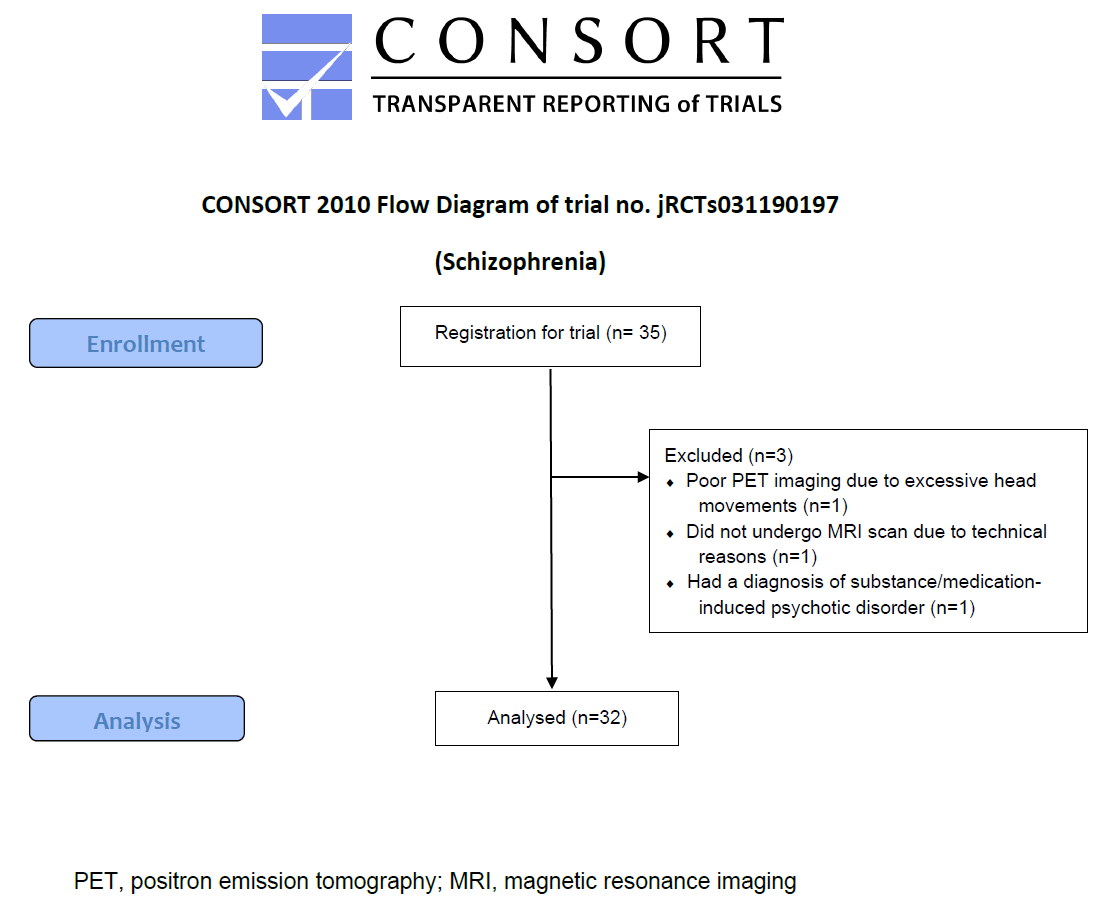


**Supplemental Fig. 1 CONSORT Chart of schizophrenia**


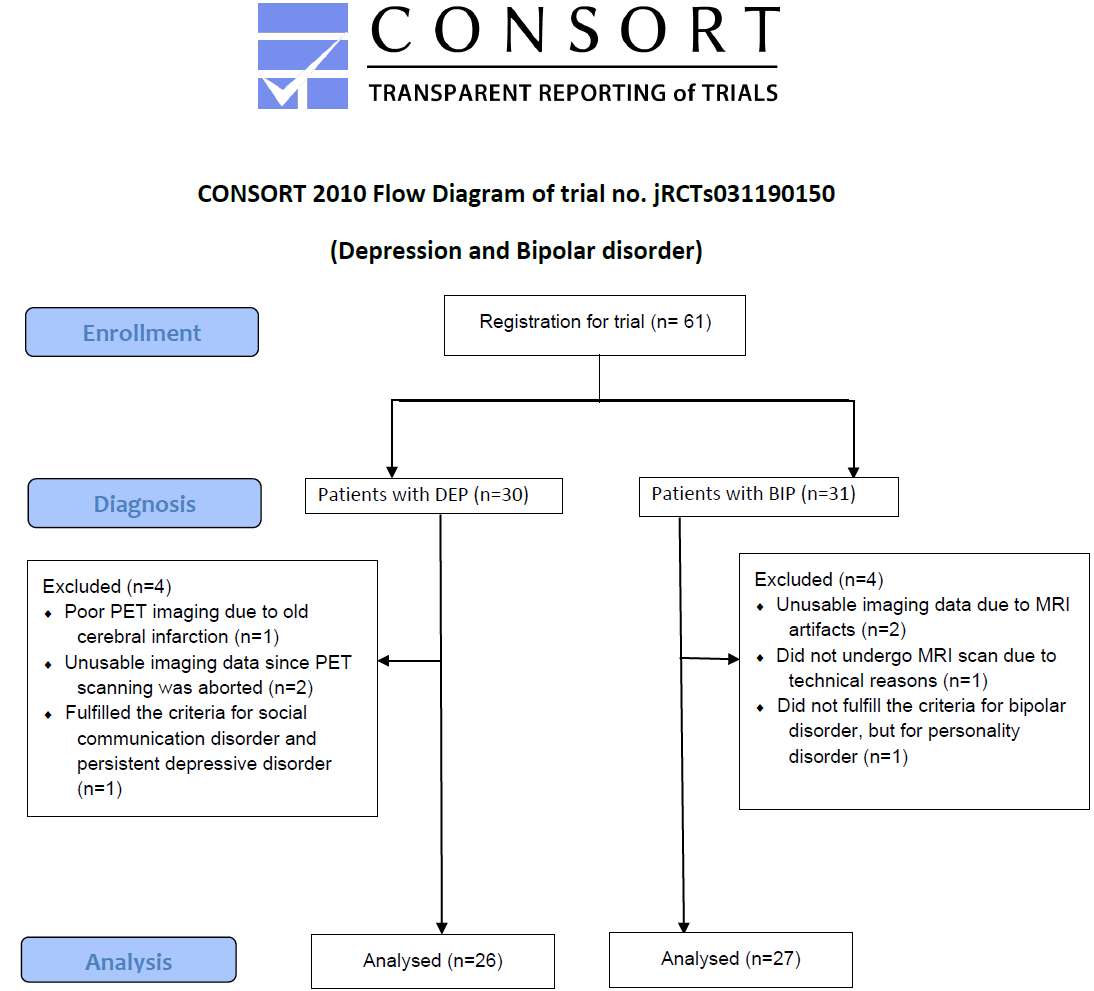


**Supplemental Fig. 2 CONSORT Chart of depression and bipolar disorder**


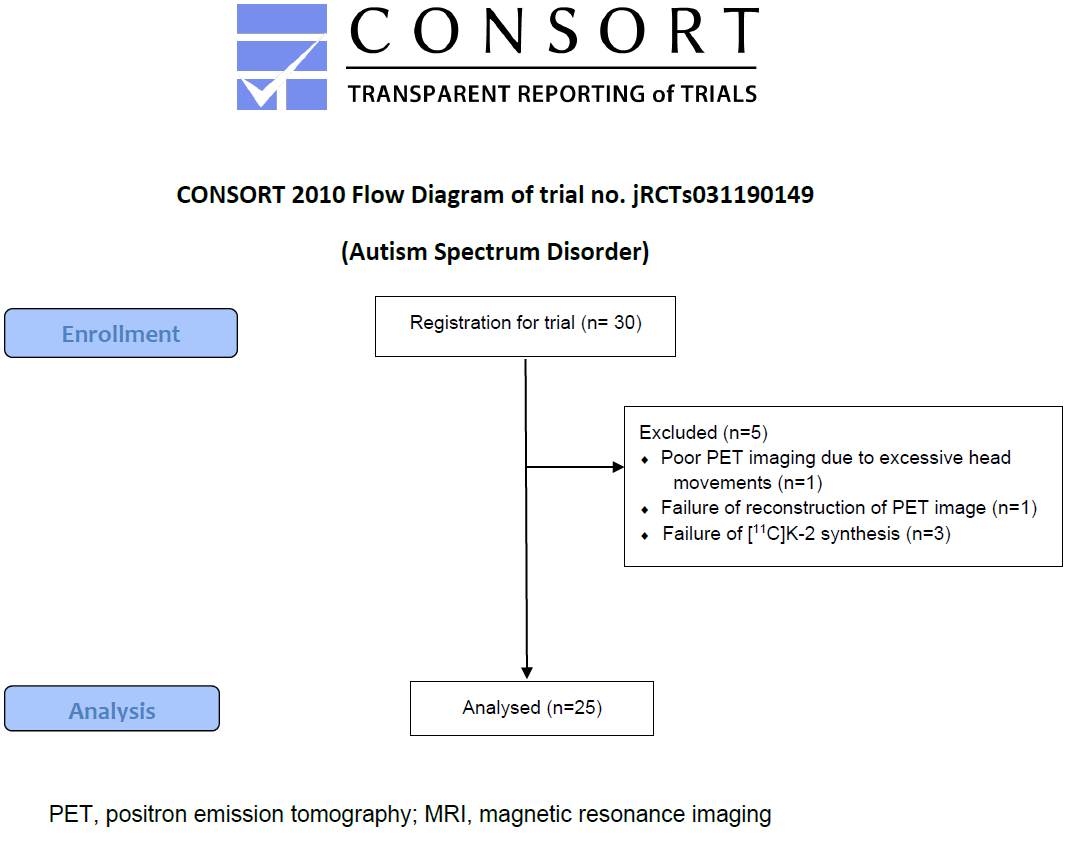


**Supplemental Fig. 3 CONSORT Chart of ASD**


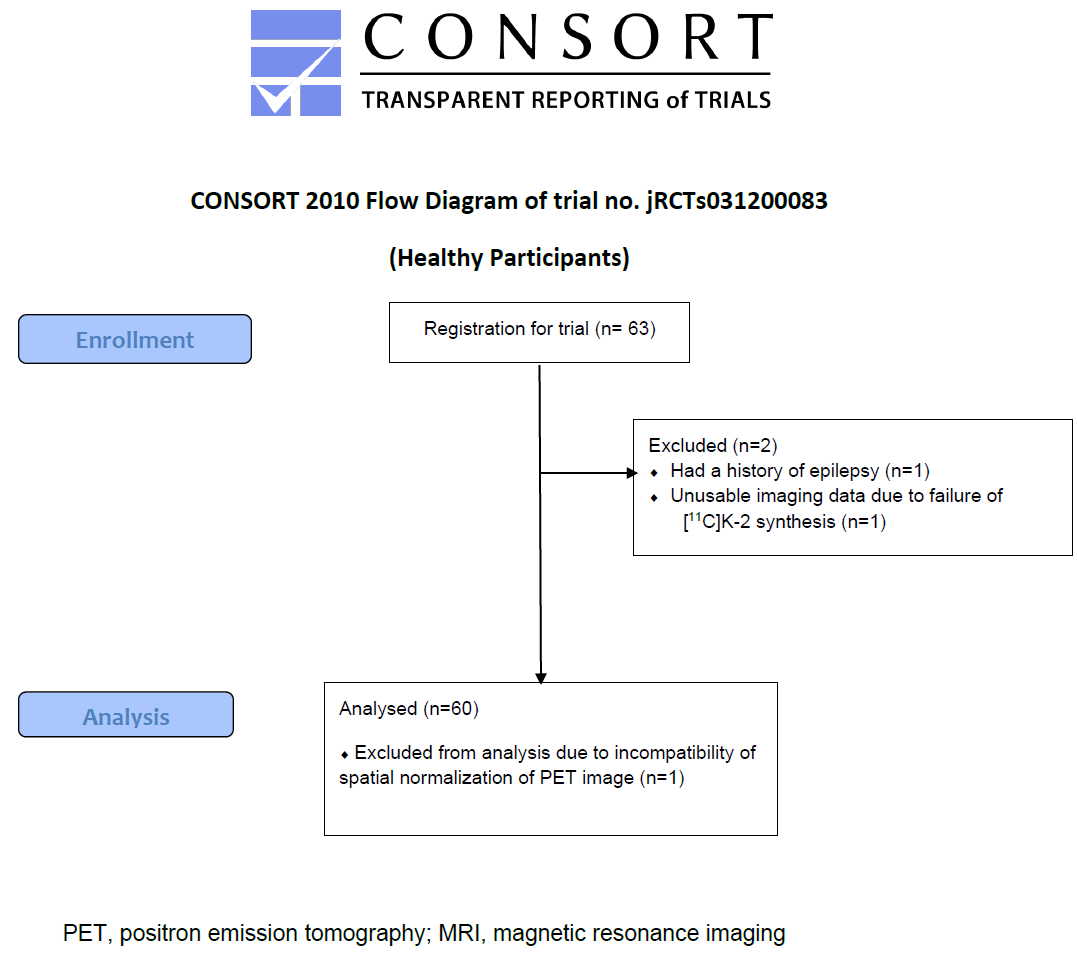


**Supplemental Fig. 4 CONSORT Chart of healthy participants**


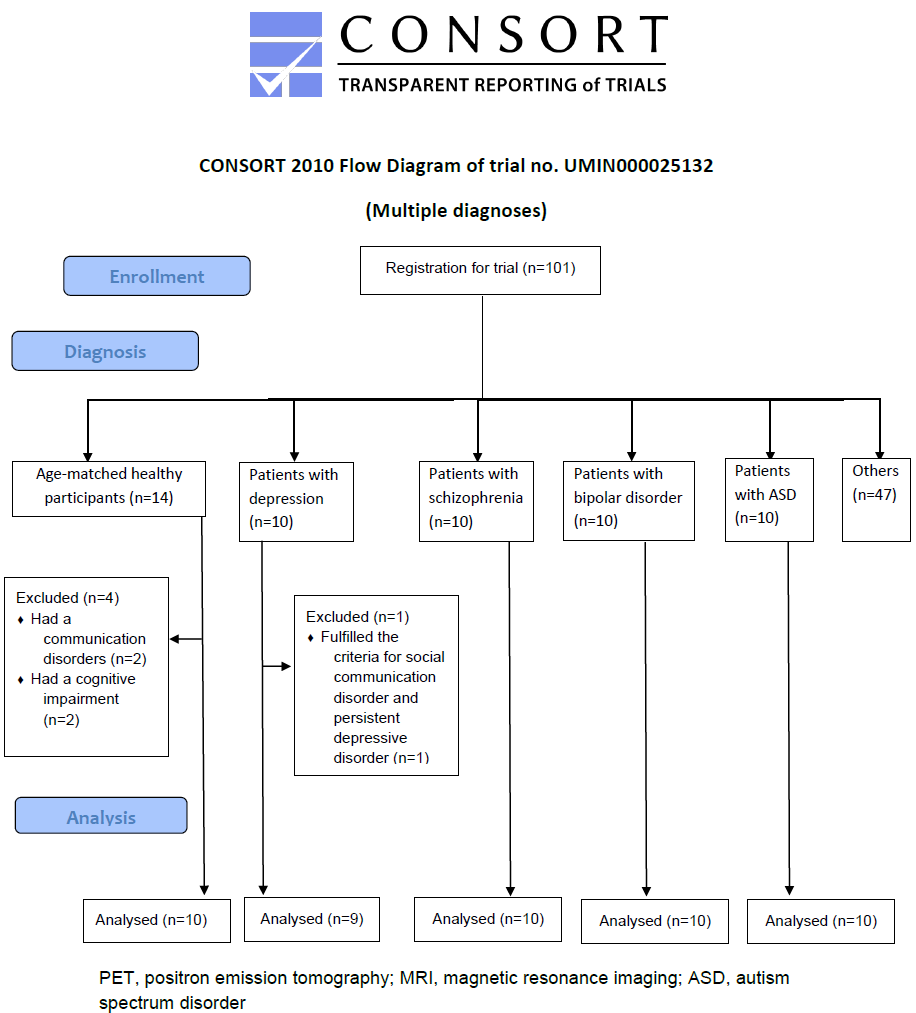


**Supplemental Fig. 5 CONSORT Chart of multiple diagnoses**

**
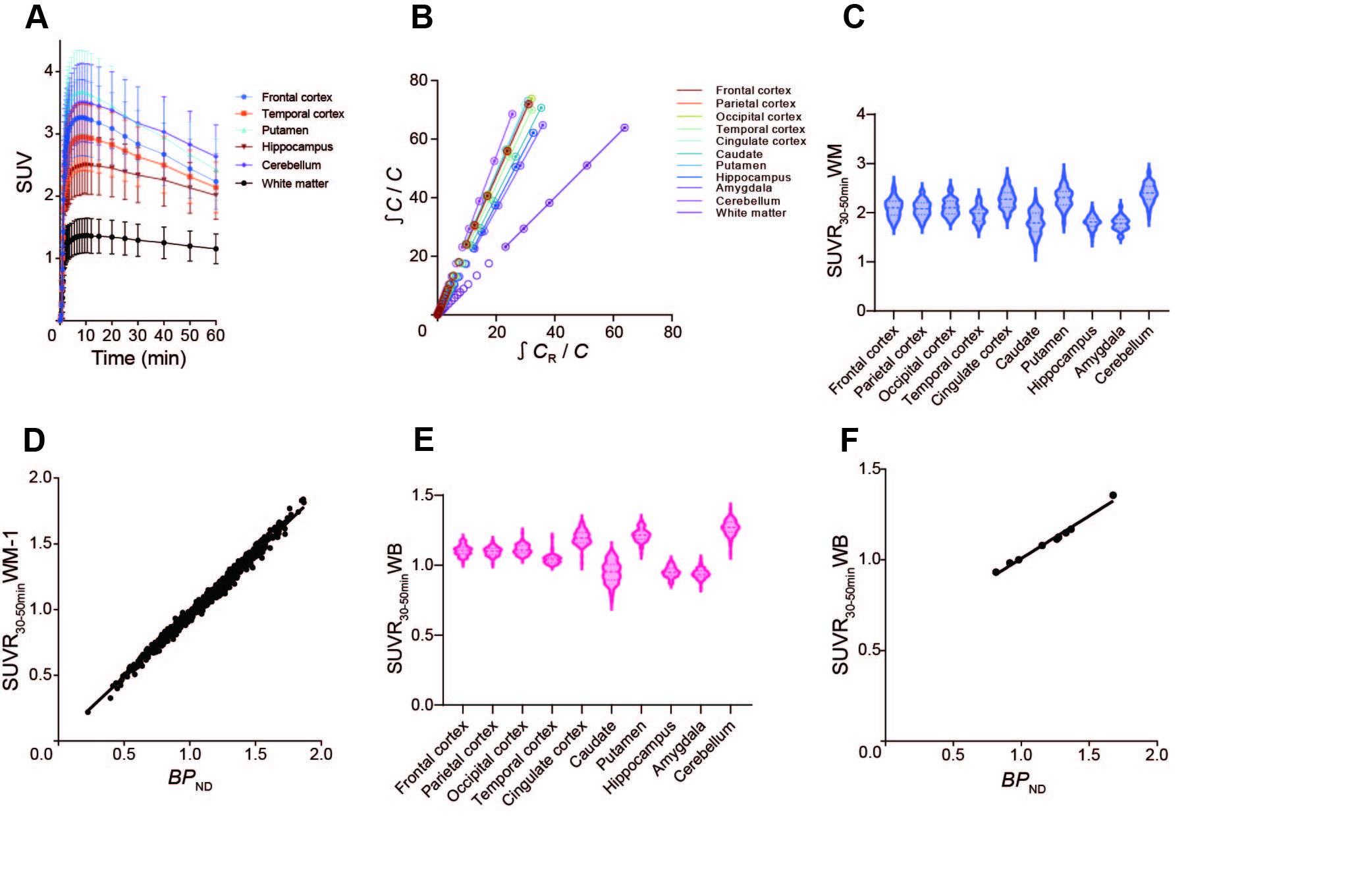
**

**Supplemental Fig. 6 Characteristics of [^11^C]K-2 in healthy participants.**

(**A**) Averaged tissue time activity curves (tTACs) in the brain regions of healthy participants (n = 70). Data are shown as the mean ± SD. (**B**) Logan graphical analysis (LGA) where the ratio between an integrated tTAC in the reference region (white matter; C_R_) and a tTAC (*C*), and an integrated *C* and *C* itself, are plotted on x- and y-axes, respectively. LGA of a representative healthy participant is presented. (**C**) Violin plot distribution of SUVR_30–50min_WM. (**D**) Correlation between SUVR_30-50min_WM-1 and *BP*_ND_ obtained from LGA in 10 brain regions in healthy participants (Pearson’s correlation analysis: correlation coefficient = 0.9944, *P* < 0.0001, Y = 0.9434*X + 0.0182). (**E**) Violin plot distribution of SUVR_30–50min_WB. (**F**) Correlation between SUVR_30-50min_WB and *BP*_ND_ obtained from LGA in 10 brain regions in a healthy participant (Pearson’s correlation analysis: correlation coefficient = 0.9912, *P* < 0.0001, Y = 0.4689*X + 0.5382).

**
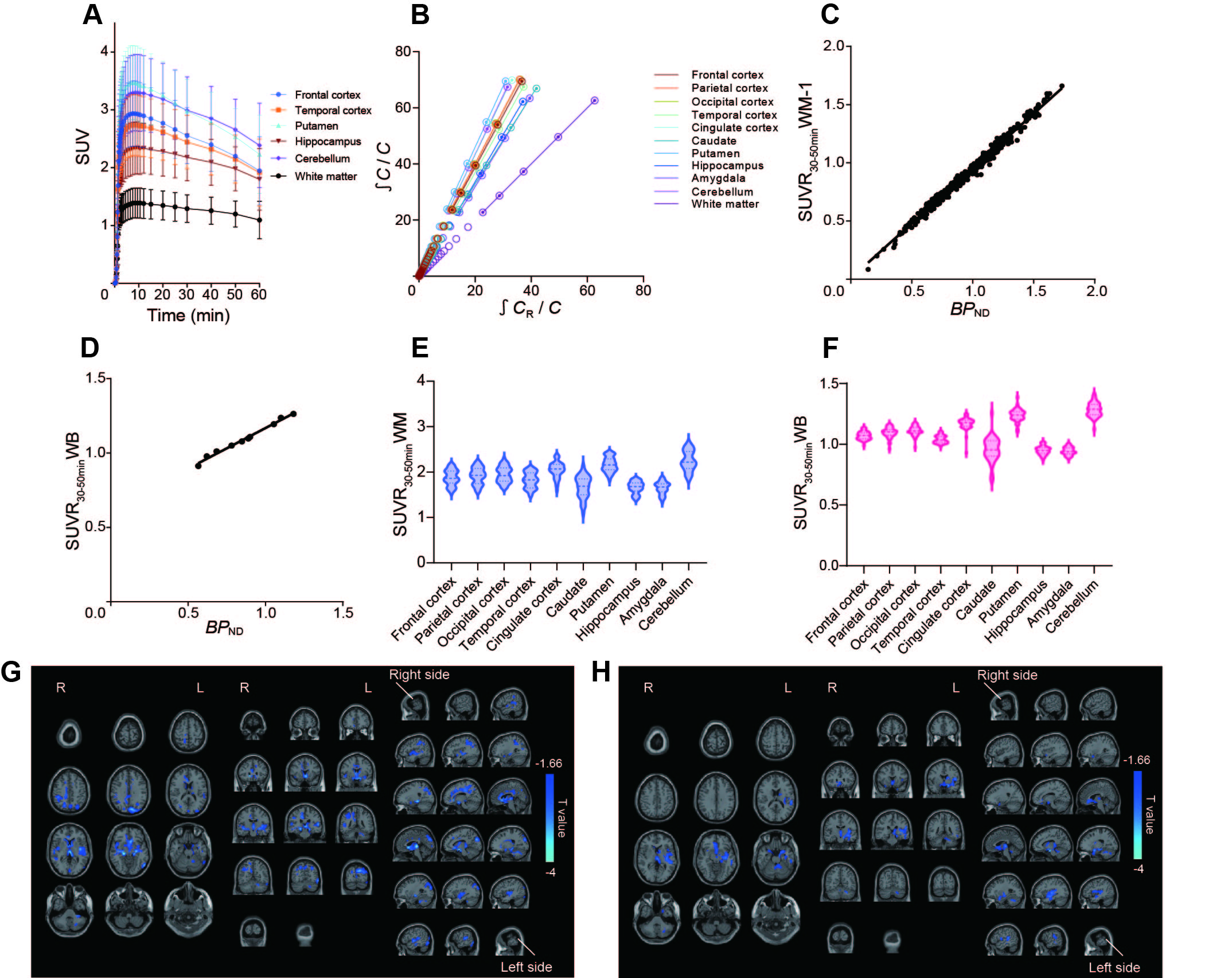
**

**Supplemental Fig. 7 Characteristics of [^11^C]K-2 and state regions in patients with schizophrenia.**

(**A**) Averaged tTACs in the brain regions of patients with schizophrenia (*n*=42). Data are shown as the mean ± SD. (**B**) LGA where the ratio between an integrated tTAC in the reference region (*C*_R_) and a tTAC (*C*), and an integrated *C* and *C* itself, are plotted on x- and y-axes, respectively. LGA of a representative patient with schizophrenia is presented. (**C**) Correlation between SUVR_30-50min_WM-1 and *BP*_ND_ obtained from LGA in 10 brain regions in patients with schizophrenia (Pearson’s correlation analysis: correlation coefficient = 0.9942, *P* < 0.0001, Y = 0.9433*X + 0.0079). (**D**) Correlation between SUVR_30-50min_WB and *BP*_ND_ obtained from LGA in 10 brain regions in a patient with schizophrenia (Pearson’s correlation analysis: correlation coefficient = 0.9946, *P* < 0.0001, Y = 0.5443*X + 0.6235). (**E**) Violin plot distribution of SUVR_30–50min_WM. (**F**) Violin plot distribution of SUVR_30–50min_WB. (**G**) Brain regions showing a significant negative correlation between SUVR_30-50min_WB and PANSS scores for positive symptoms in patients with schizophrenia (*P* < 0.05, T < -1.68, one-tailed, FDRc) (Brain-wide presentation). (**H**) Brain regions showing a significant negative correlation between SUVR_30-50min_WB and PANSS scores for negative symptoms in patients with schizophrenia (*P* < 0.05, T < -1.68, one-tailed, FDRc) (Brain-wide presentation).

**
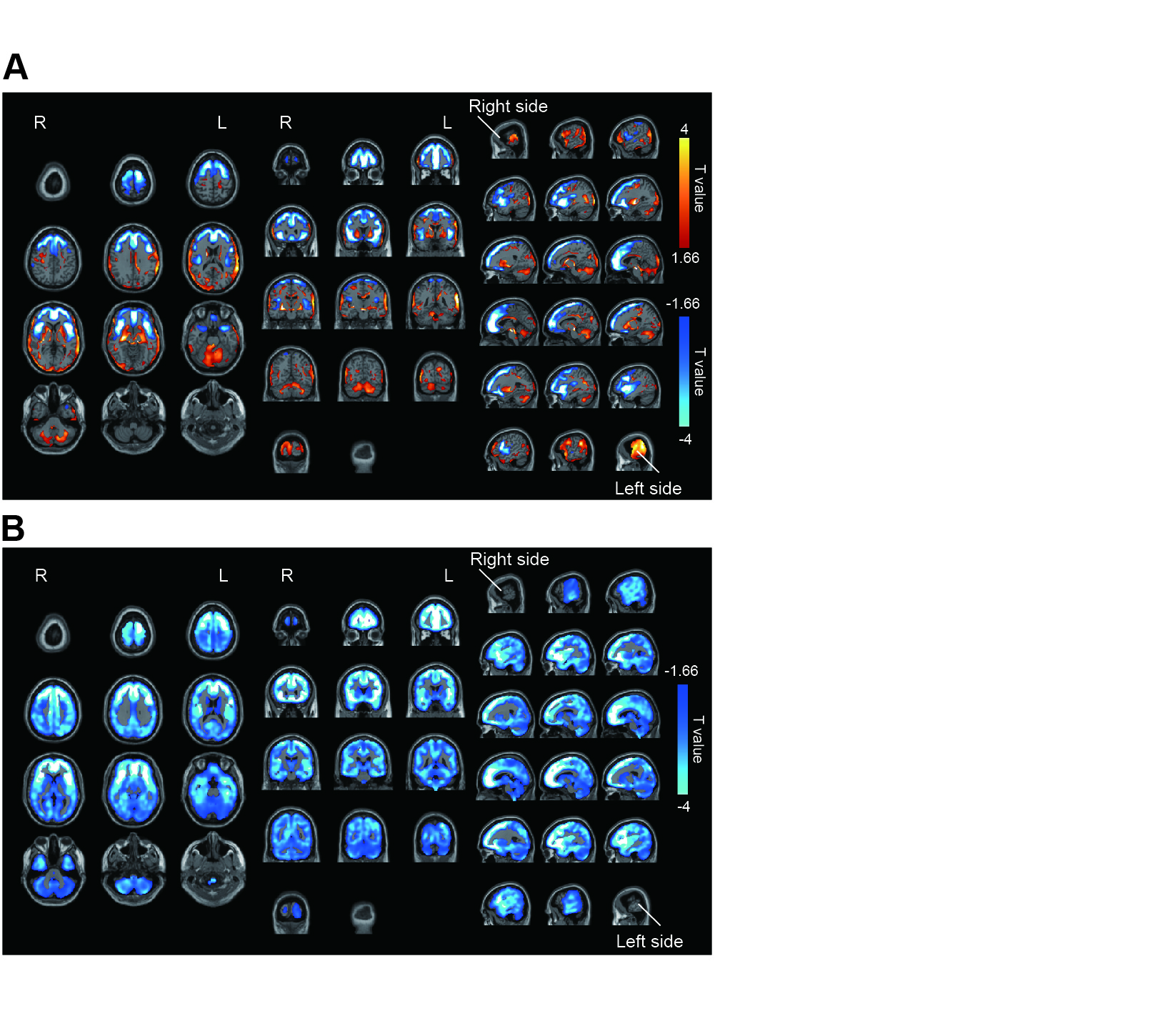
**

**Supplemental Fig. 8 Altered AMPAR distribution in patients with schizophrenia compared with healthy participants.**

(**A**) Increase (red) and reduction (blue) of SUVR_30-50min_WB in patients with schizophrenia compared to healthy participants (*P* < 0.05, increase of SUVR_30-50min_WB: T > 1.66, reduction of SUVR_30-50min_WB: T < -1.66, one-tailed, FDRc) (Brain-wide presentation). (**B**) Reduction (blue) of SUVR_30-50min_WM in patients with schizophrenia compared to healthy participants (*P* < 0.05, T < -1.66, one-tailed, FDRc) (Brain-wide presentation).

**
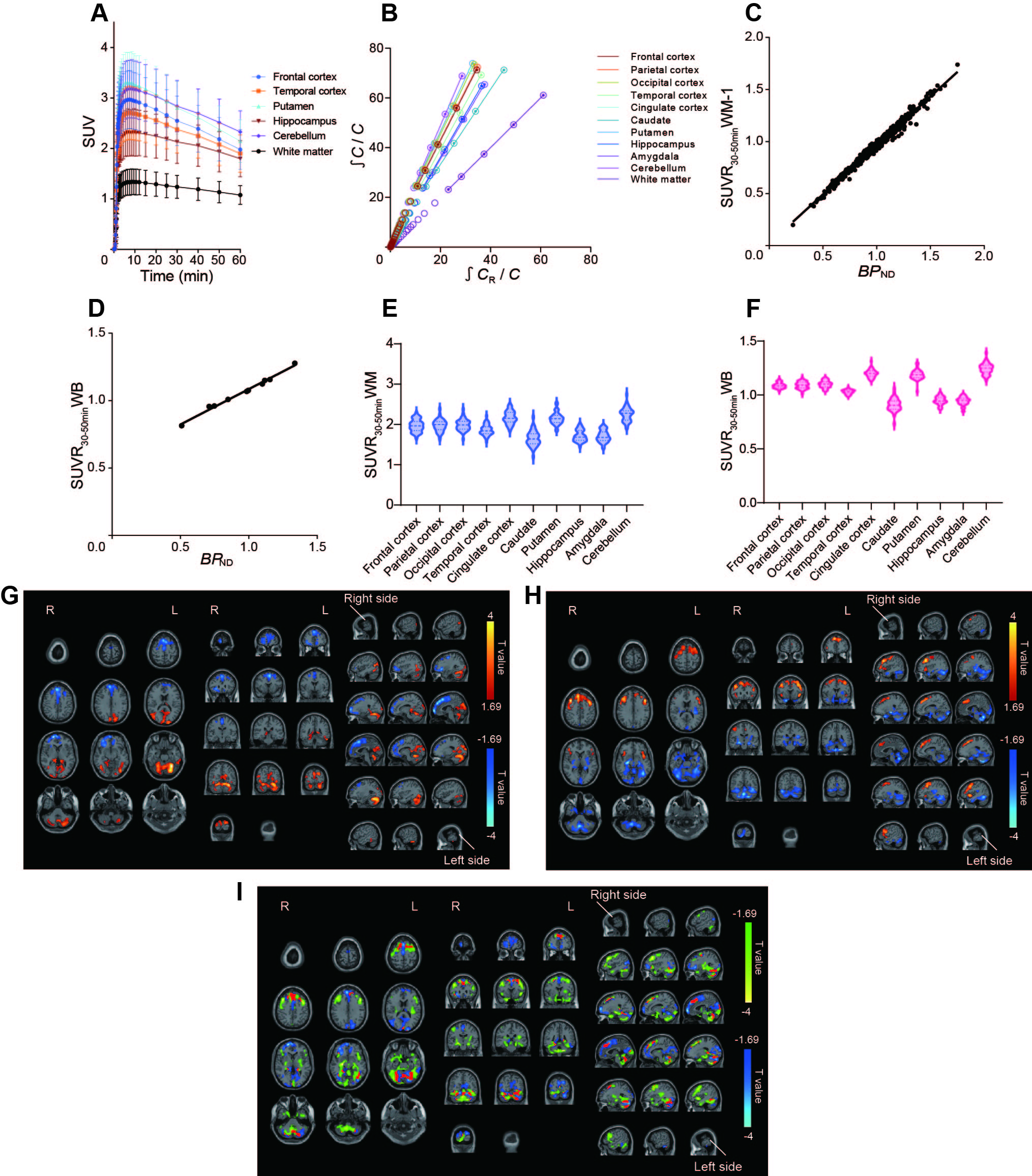
**

**Supplemental Fig. 9 Characteristics of [^11^C]K-2 and state regions in patients with bipolar disorder.**

(**A**) Averaged tTACs in the brain regions of patients with bipolar disorder (*n*=37). Data are shown as the mean ± SD. (**B**) LGA where the ratio between an integrated tTAC in the reference region (*C*_R_) and a tTAC (*C*), and an integrated *C* and *C* itself, are plotted on x- and y-axes, respectively. LGA of a representative patient with bipolar disorder is presented. (**C**) Correlation between SUVR_30-50min_WM-1 and *BP*_ND_ obtained from LGA in 10 brain regions in patients with bipolar disorder (Pearson’s correlation analysis: correlation coefficient = 0.9936, *P* < 0.0001, Y = 0.9408*X + 0.02121). (**D**) Correlation between SUVR_30-50min_WB and *BP*_ND_ obtained from LGA in a patient with bipolar disorder (Pearson’s correlation analysis: correlation coefficient = 0.9953, *P* < 0.0001, Y =0.5252*X + 0.5604). (**E**) Violin plot distribution of SUVR_30–50min_WM. (**F**) Violin plot distribution of SUVR_30–50min_WB. (**G**) Brain regions showing a significant positive (red) and negative (blue) correlation between SUVR_30-50min_WB and the 17-item HAM-D scores in patients with bipolar disorder (*P* < 0.05, positive correlation: T > 1.69, negative correlation: T < -1.69, one-tailed, FDRc) (Brain-wide presentation). (**H**) Brain regions showing a significant positive (red) and negative (blue) correlation between SUVR_30-50min_WB and YMRS scores in patients with bipolar disorder (*P* < 0.05, positive correlation: T > 1.69, negative correlation: T < -1.69, one-tailed, FDRc) (Brain-wide presentation). (**I**) Brain regions showing a significant correlation between SUVR_30-50min_WB and the 17-item HAM-D score (blue) or YMRS score (green) in patients with bipolar disorder (*P* < 0.05, one-tailed, FDRc). Red regions show where the two regions overlap (Brain-wide presentation).

**
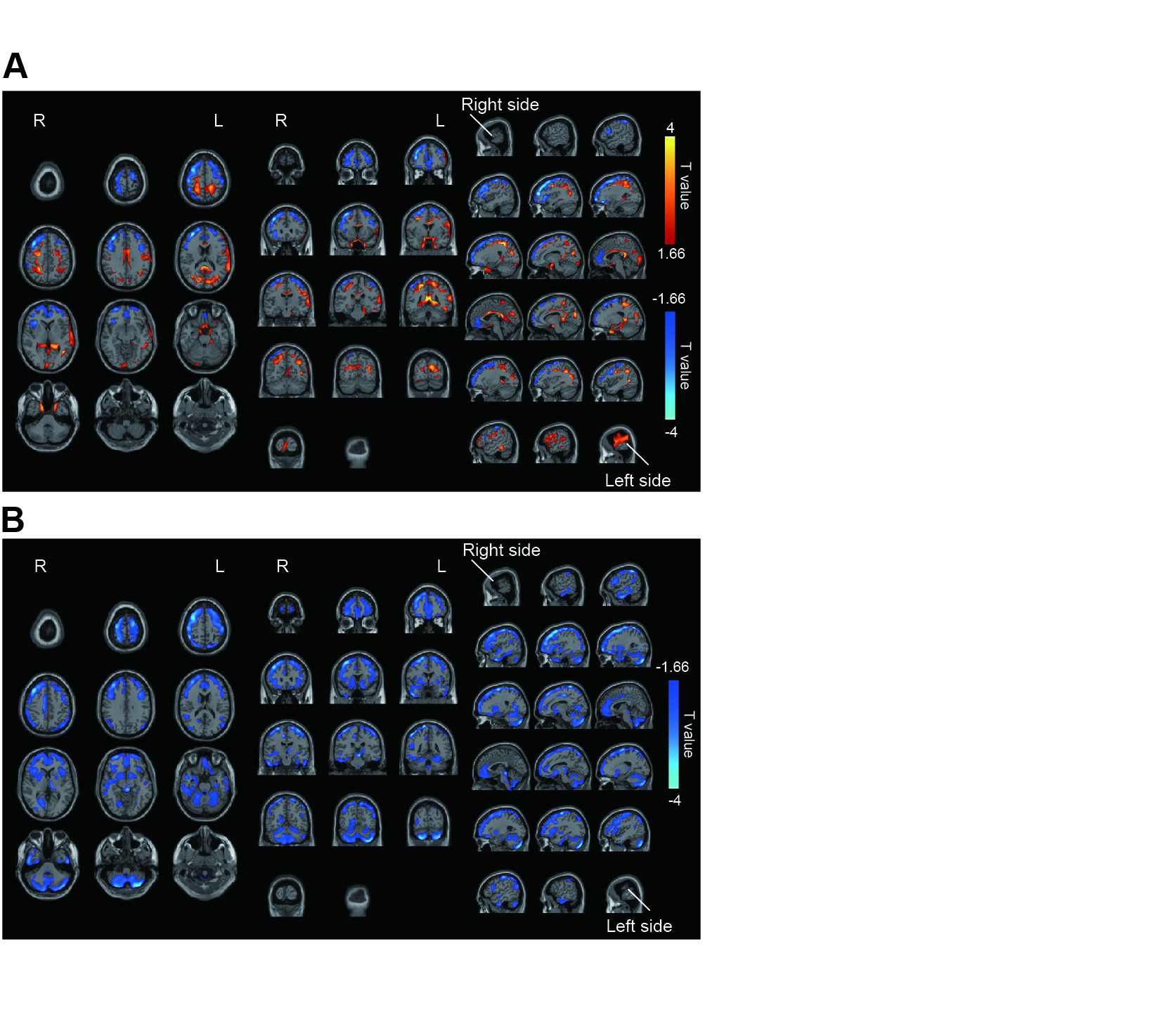
**

**Supplemental Fig. 10 Altered AMPAR distribution in patients with bipolar disorder compared with healthy participants.**

(A) Relative increase (red) and reduction (blue) of SUVR_30-50min_WB in patients with bipolar disorder compared to healthy subjects (*P* < 0.05, increase of SUVR_30-50min_WB: T > 1.66, reduction of [^11^C]K-2 retention: T < -1.66, one-tailed, FDRc) (Brain-wide presentation). (B) Reduction (blue) of SUVR_30-50min_WM in patients with bipolar disorder compared to healthy participants (*P* < 0.05, T < -1.66, one-tailed, FDRc) (Brain-wide presentation).

**
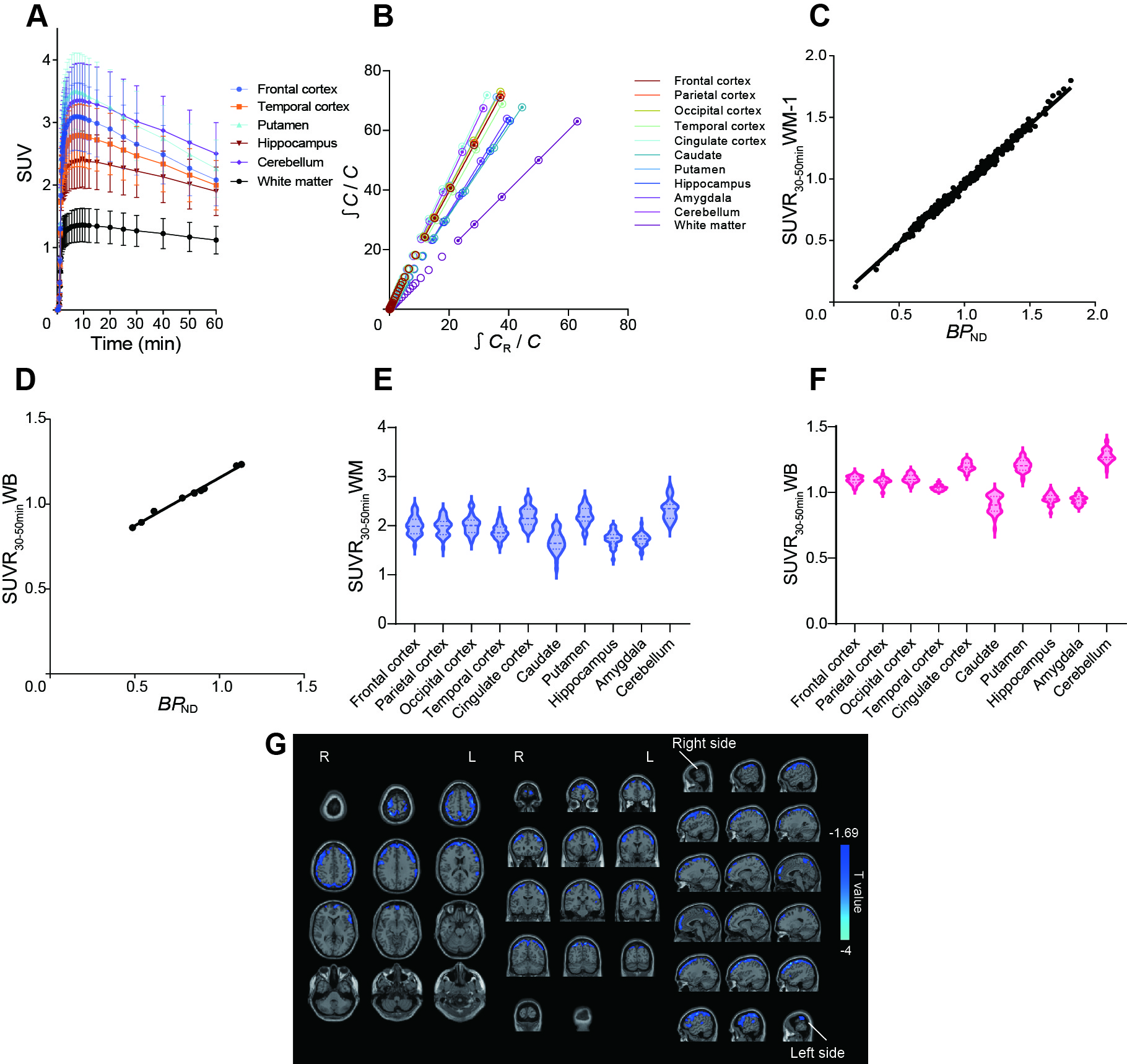
**

**Supplemental Fig. 11 Characteristics of [^11^C]K-2 and state regions in patients with depression.**

(**A**) Averaged tTACs in the brain regions of patients with depression (*n*=35). Data are shown as the mean ± SD. (**B**) LGA where the ratio between an integrated tTAC in the reference region (*C*_R_) and a tTAC (*C*), and an integrated *C* and *C* itself, are plotted on x- and y-axes, respectively. LGA of a representative patient with depression is presented. (**C**) Correlation between SUVR_30-50min_WM-1 and *BP*_ND_ obtained from LGA in 10 brain regions in patients with depression (Pearson’s correlation analysis: correlation coefficient = 0.9959, *p* < 0.0001, Y = 0.9617*X - 0.0026). (**D**) Correlation between SUVR_30-50min_WB and *BP*_ND_ obtained from LGA in 10 brain regions in a patient with depression (Pearson’s correlation analysis: correlation coefficient=0.9957, *P* < 0.0001, Y = 0.5650*X + 0.5900). (**E**) Violin plot distribution of SUVR_30–50min_WM. (**F**) Violin plot distribution of SUVR_30–50min_WB. (**G**) Brain regions showing a significant negative correlation between SUVR_30-50min_WB and the 17-item HAM-D scores in patients with depression (*P* < 0.05, T < -1.69, one-tailed, FDRc) (Brain-wide presentation).


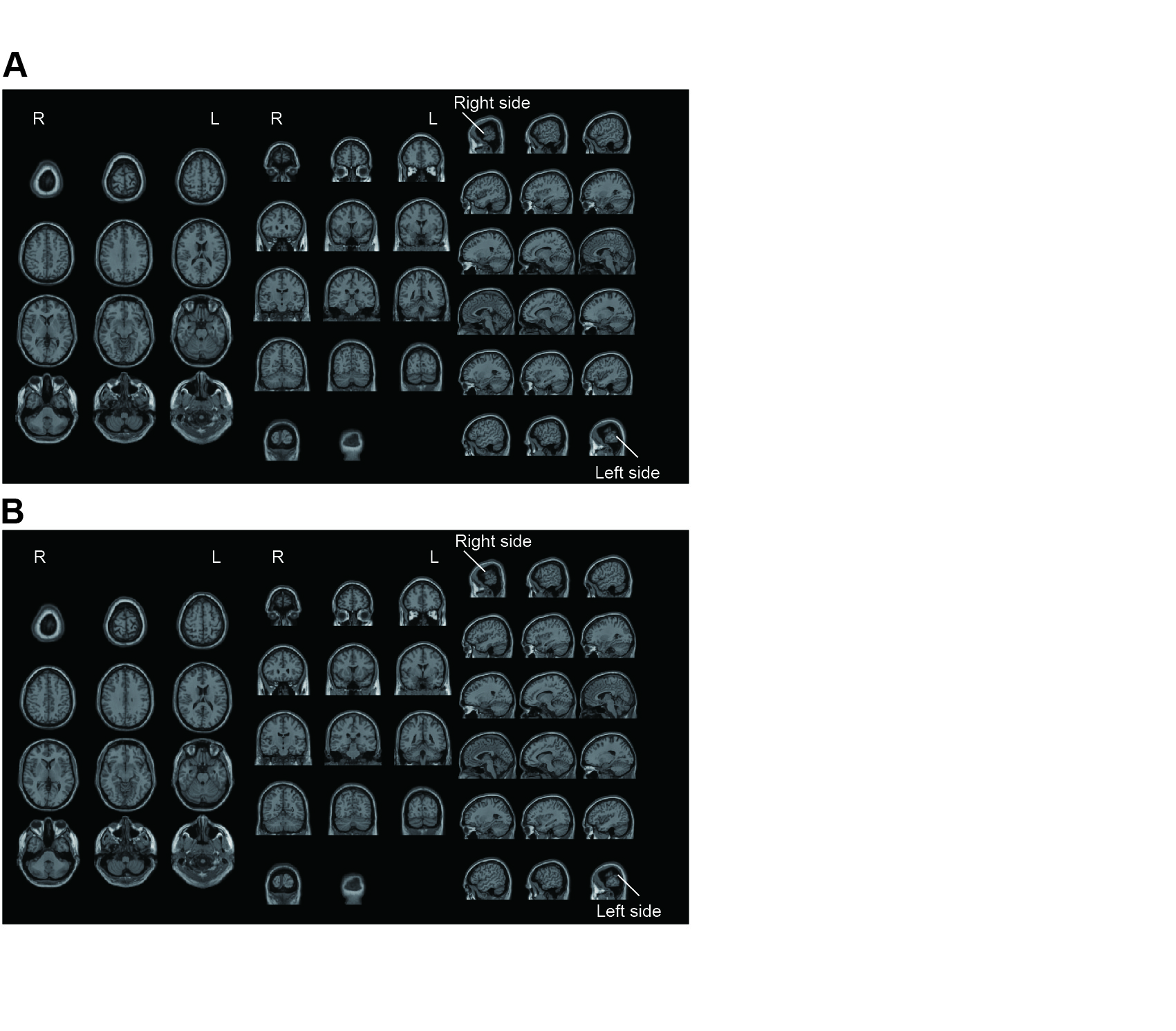


**Supplemental Fig. 12 Comparison of SUVR_30-50min_ between patients with depression and healthy participants.**

(**A**) No significant difference of SUVR_30-50min_WB between patients with depression and healthy participants. (**B**) No significant difference of SUVR_30-50min_WM between patients with depression and healthy participants.

**
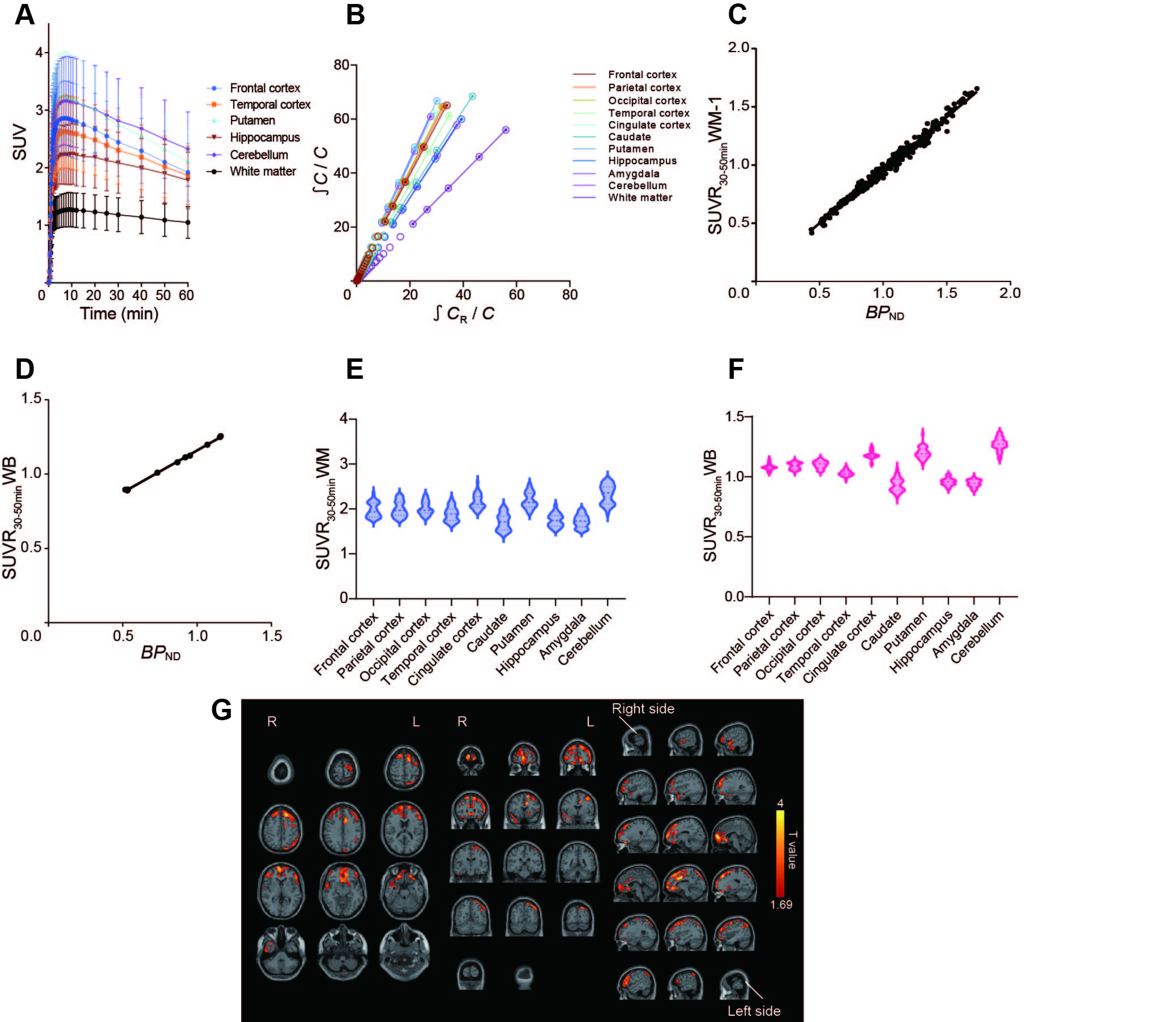
**

**Supplemental Fig. 13 Characteristics of [^11^C]K-2 and state regions in patients with ASD.**

(**A**) Averaged tTACs in the brain regions of patients with ASD (*n*=35). Data are shown as the mean ± SD. (**B**) LGA where the ratio between an integrated tTAC in the reference region (*C*_R_) and a tTAC (*C*), and an integrated *C* and *C* itself, are plotted on x- and y-axes, respectively. LGA of a representative patient with ASD is presented. (**C**) Correlation between SUVR_30-50min_WM-1 and *BP*_ND_ obtained from LGA in 10 brain regions in patients with ASD (Pearson’s correlation analysis: correlation coefficient = 0.9934, *P* < 0.0001, Y = 0.9135*X - 0.0431). (**D**) Correlation between SUVR_30-50min_WB and *BP*_ND_ obtained from LGA in 10 brain regions in a patient with ASD (Pearson’s correlation analysis: correlation coefficient=0.9994, *P* < 0.0001, Y =0.5653*X + 0.5941). (**E**) Violin plot distribution of SUVR_30–50min_WM. (**F**) Violin plot distribution of SUVR_30–50min_WB. (**G**) Brain regions showing a significant positive correlation between SUVR_30-50min_WB and ADOS-2 Module 4 calibrated severity score in patients with ASD (*P* < 0.05, T > 1.69, one-tailed, FDRc) (Brain-wide presentation).

**
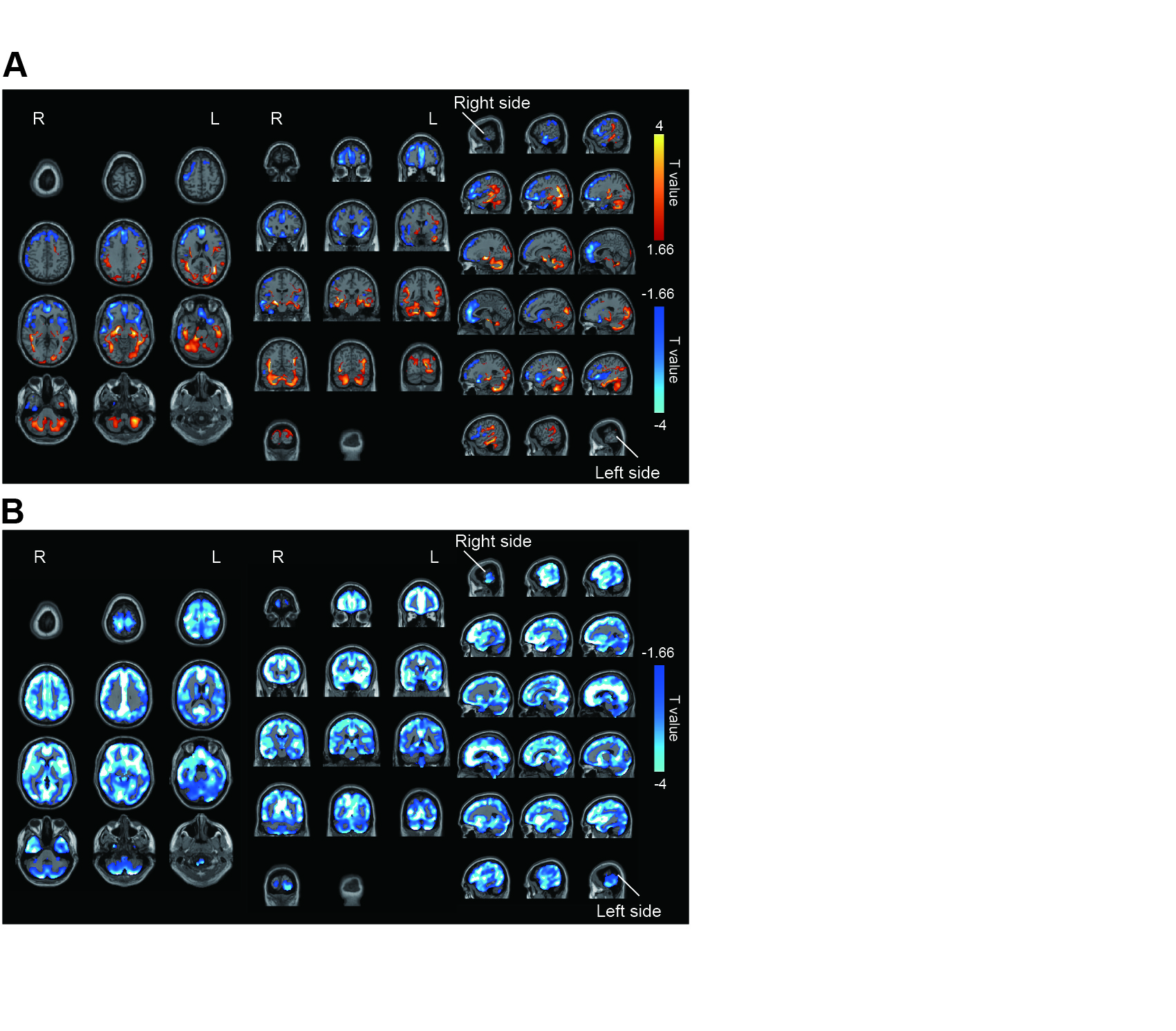
**

**Supplemental Fig. 14 Altered AMPAR distribution in patients with ASD compared with healthy participants.**

(**A**) Relative increase (red) and reduction (blue) of SUVR_30-50min_WB in patients with ASD compared to healthy subjects (*P* < 0.05, increase of SUVR_30-50min_WB: T > 1.66, reduction of [^11^C]K-2 retention: T < -1.66, one-tailed, FDRc) (Brain-wide presentation). (**B**) Reduction (blue) of SUVR_30-50min_WM in patients with ASD compared to healthy participants (*p* < 0.05, T < -1.66, one-tailed, FDRc) (Brain-wide presentation).

**
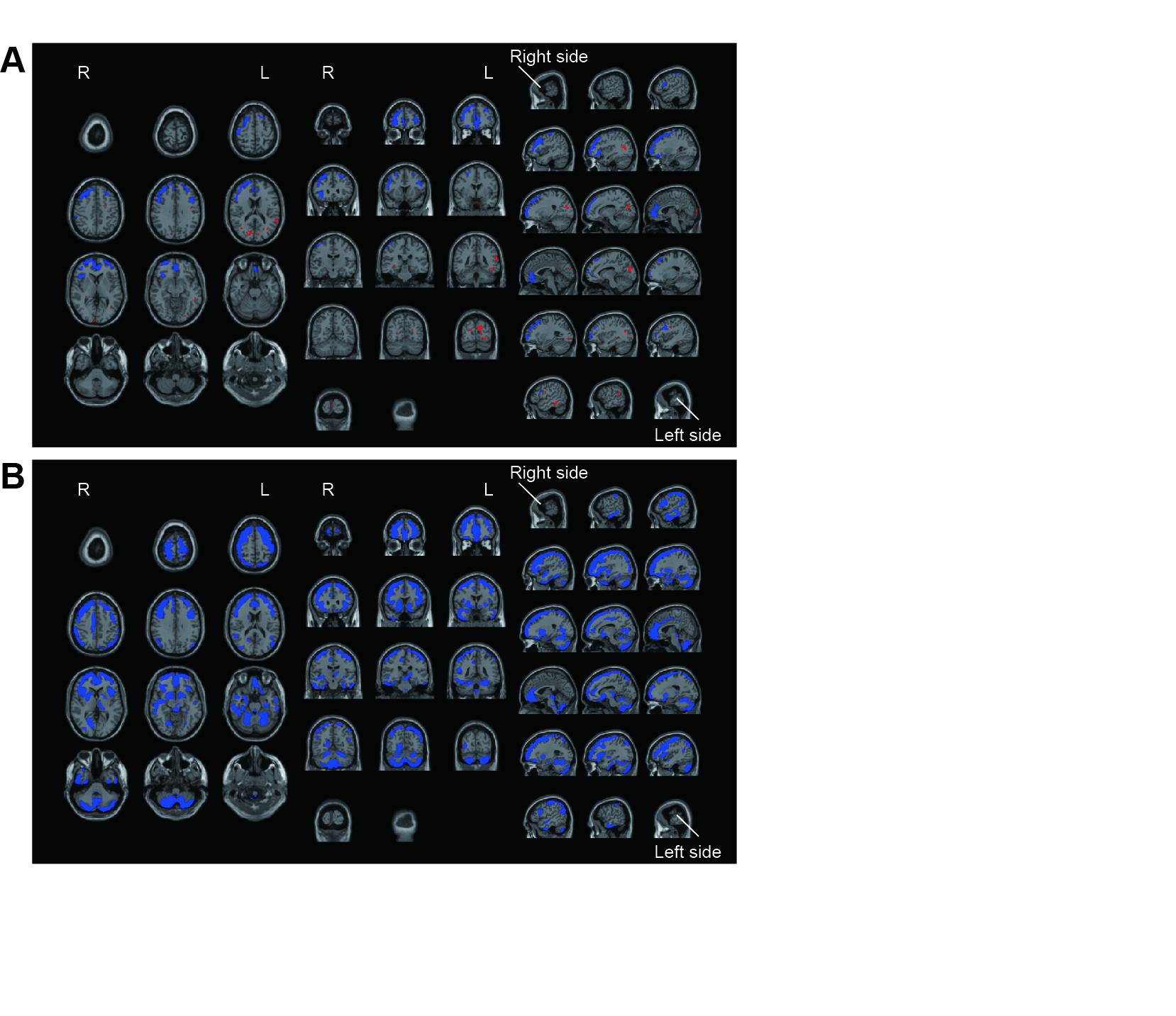
**

**Supplemental Fig. 15 Commonly affected areas across psychiatric disorders.**

(**A**) Overlapped brain regions reduced (blue) and increased (red) SUVR_30-50min_WB across in patients with schizophrenia, bipolar disorder and ASD compared with healthy participants (brain-wide presentation). (**B**) Overlapped brain regions reduced (blue) SUVR_30-50min_WM across in patients with schizophrenia, bipolar disorder and ASD compared with healthy participants (Brain-wide presentation).

**Supplemental Table 1. Demographic and Clinical Characteristics of Patients with Schizophrenia.**

| No. | Age, years | Sex | DOI, years | PANSS scores, T/P/N | Medications prescribed, mg/day, Psychotropic drugs are written in italics |
| --- | --- | --- | --- | --- | --- |
| 1 | 43 | M | 16 | 46/8/13 | *aripiprazole 24, sodium valproate 400, duloxetine 60* |
| 2 | 45 | M | 25 | 60/12/17 | *risperidone 2.5, nitrazepam 5* |
| 3 | 45 | M | 28 | 70/24/17 | *olanzapine 20* |
| 4 | 41 | M | 17 | 90/27/25 | *olanzapine 20* |
| 5 | 39 | M | 12 | 65/13/19 | *risperidone 12, levomepromazine 200, lithium bicarbonate 300, lorazepam 3, nitrazepam 10, biperiden 3*, magnesium oxide 330 |
| 6 | 40 | M | 19 | 57/16/17 | *fluphenazine 6, miltazapine 30, duloxetine 40, clonazepam 2, flunitrazepam 2, biperiden 4*, ursodeoxycholic acid 200, fenofibrate 80, febuxostat 20 |
| 7 | 39 | M | 7 | 51/9/17 | *risperidone 2, flunitrazepam 2, nitrazepam 10, clonazepam 0.5* |
| 8 | 45 | M | 21 | 64/13/25 | *asenapine 20, clonazepam 0.5*, linagliptin 5, rosuvastatin2.5 |
| 9 | 47 | M | 21 | 45/9/10 | *olanzapine 10, risperidone 0.5* |
| 10 | 47 | M | 2 | 69/20/19 | *aripiprazole 12* |
| 11 | 45 | F | 21 | 105/26/27 | *lithium 400, clonazepam 3.5, olanzapine 20, risperidone 6, biperiden 1*, magnesium oxide 1000, pantethine 400 |
| 12 | 30 | M | 14 | 53/12/20 | *blonanserin 16* |
| 13 | 49 | F | 24 | 80/21/18 | *brexpiprazole 2, pregabalin 150, duloxetine 40, olanzapine 5, trazodon 50*, flavin adenine dinucleotide sodium 30, pyridoxal phosphate hydrate 60, bepotastine 20 |
| 14 | 40 | M | 7 | 91/24/22 | *clozapine 300*, magnesium oxide 830, ethyl icosapentate 2700, ascorbic acid 750, montelukast sodium 10, pitavastatin calcium hydrate 1, edoxaban tosilate hydrate 60 |
| 15 | 45 | F | 15 | 55/28/7 | *brexpiprazole 1* |
| 16 | 38 | M | 19 | 94/26/22 | *suvorexant 20, sodium valproate 800, flunitrazepam 2, aripiprazole long-acting injection 400** |
| 17 | 29 | F | 5 | 80/22/18 | None |
| 18 | 31 | M | 5 | 158/34/40 | *aripiprazole long-acting injection 400** |
| 19 | 26 | M | 2 | 75/15/21 | *paliperidone long-acting injection 75** |
| 20 | 28 | F | 8 | 56/11/13 | *clozapine 350, lithium 200, escitalopram 10, clonazepam 2.5, biperiden 2, gabapentin enacarbil 300* |
| 21 | 33 | M | 7 | 85/25/22 | *olanzapine 15* |
| 22 | 50 | M | 22 | 53/15/18 | *olanzapine 20* |
| 23 | 39 | M | 11 | 64/14/18 | *olanzapine 20, aripiprazole 12* |
| 24 | 25 | F | 10 | 76/21/15 | *clozapine 500, lithium 600, sodium valproate 400, clonazepam 1* |
| 25 | 20 | M | 2 | 86/19/24 | *olanzapine 20* |
| 26 | 26 | M | 5 | 102/22/25 | *olanzapine 12.5* |
| 27 | 45 | M | 15 | 55/7/24 | *brotizolam 0.25, flunitrazepam 1, nitrazepam 1, aripiprazole 12, sertraline 100, milnacipran 50, arotinolol 20, icosapentate 1800, pemafibrate 0.2, ezetimibe 10* |
| 28 | 58 | M | 38 | 108/23/27 | *olanzapine 15, asenapine 5, sodium valproate 1000, suvorexant 20, trazodon 50, duloxetine 60,* acetaminophen 400, methotrexate 8, bucillamine 15, folic acid 15, trihexyphenidyl hydrochloride 8, mexiletine 9, magnesium oxide 2000, olopatadine hydrochloride 10, sennoside 24, vonoprazan fumarate 10, iguratimod 50, polycarbophil calcium 3000, elobixibat hydrate 10, bifidobacterium 3, Tramadol hydrochloride, acetaminophen 8, |
| 29 | 24 | F | 5 | 85/18/21 | *brexpiprazole 2,* levocetirizine 10, magnesium oxide 500, picosulfate 5, elobixibat 10 |
| 30 | 39 | F | 17 | 100/24/25 | *aripiprazole long-acting injection 300** |
| 31 | 54 | M | 6 | 43/7/17 | *paliperidone long-acting injection 100** |
| 32 | 37 | F | 11 | 54/14/14 | *aripiprazole 24, clonazepam 1* |
| 33 | 35 | M | 9 | 67/15/14 | *brexpiprazole 1, quetiapine 25* |
| 34 | 50 | M | 5 | 89/15/37 | *risperidone 6, nitrazepam 10* |
| 35 | 50 | M | 29 | 49/9/18 | *risperidone 4, nitrazepam 10, quetiapine 100, trazodone 100, biperiden 2,* sennoside 24, propiverine hydrochloride 20 |
| 36 | 27 | F | 2 | 53/12/16 | *clozapine 175, lithium 400*, sennoside 36, elobixibat hydrate 10, linaclotide 0.25, minocycline 100 |
| 37 | 45 | F | 30 | 105/32/20 | *risperidone 6, sodium valproate 1000, lorazepam 2, biperiden 2* |
| 38 | 38 | M | 6 | 98/22/27 | *brexpiprazole 1.5, promethazine 25* |
| 39 | 26 | M | 7 | 74/15/18 | *aripiprazole long-acting injection 400, quetiapine 25* |
| 40 | 33 | M | 6 | 81/19/19 | *olanzapine 10, ramelteon 8* |
| 41 | 40 | F | 5 | 60/14/17 | *aripiprazole 3, quetiapine 25, ramelteon 8*, magnesium oxide 250 |
| 42 | 49 | M | 32 | 135/33/40 | *clozapine 400*, magnesium oxide 1000 |

M, male; F, female; DOI, duration of illness; PANSS, Positive and Negative Syndrome Scale; T, total; P, positive scale; N, negative scale

* Given bimonthly

**Supplemental Table 2. Demographic and Clinical Characteristics of Patients with bipolar disorder.**

| No. | Age, years | Sex | DOI, years | HAMD-17 total score | YMRS | Medications prescribed, mg/day, Psychotropic drugs are written in italics |
| --- | --- | --- | --- | --- | --- | --- |
| 1 | 38 | M | 11 | 10 | 5 | *lithium 600* |
| 2 | 46 | M | 16 | 0 | 2 | *lithium 1200, duloxetine 60, aripiprazole 3* |
| 3 | 46 | M | 3 | 1 | 0 | *lithium 1000, lamotrigine 400, quetiapine 50, zolpidem 10, clonazepam 1.5, mirtazapine 45, triazolam 0.25, notriptyline 75* |
| 4 | 44 | M | 15 | 1 | 0 | *lithium 1000* |
| 5 | 39 | M | 11 | 2 | 12 | *lithium 1200, lamotrigine 100* |
| 6 | 35 | M | 10 | 11 | 2 | *lithium 1200* |
| 7 | 47 | M | 12 | 23 | 0 | *lithium 600, escitalopram 10, etizolam 0.5, quazepam 20*, polycarbophil calcium 1500 |
| 8 | 44 | M | 27 | 10 | 17 | *lithium 1400, quetiapine 150* |
| 9 | 41 | M | 18 | 9 | 7 | *lithium 800, lamotrigine 200, aripiprazole 3, levomepromazine 10, suvorexant 20, ethyl loflazepate 1* |
| 10 | 44 | M | 22 | 3 | 22 | None |
| 11 | 50 | M | 15 | 0 | 9 | *lithium 1000, aripiprazole 6, ethyl loflazepate 2, mirtazapine 30, clomipramine 150* |
| 12 | 26 | M | 9 | 0 | 10 | *lithium 600* |
| 13 | 40 | M | 16 | 5 | 22 | *lithium 600, suvorexant 20, lunitrazepam 2, ramelteon 8, risperidone 1, nitrazepam 5* |
| 14 | 39 | F | 19 | 6 | 0 | *lithium 600, trazodone 200, brotizolam 0.25* |
| 15 | 32 | M | 17 | 0 | 3 | *quetiapine 200, mirtazapine 15, clonazepam 1* |
| 16 | 29 | F | 10 | 15 | 4 | *lithium 900, aripiprazole 12, levocetirizine 10*, vonoprazan fumarate 10 |
| 17 | 54 | F | 22 | 6 | 21 | *lithium 600, amlodipine besilate 5, pitavastatin calcium hydrate 2* |
| 18 | 44 | M | 29 | 1 | 9 | *lithium 200* |
| 19 | 43 | M | 26 | 10 | 4 | *lithium 600, lamotrigine 50* |
| 20 | 32 | M | 14 | 1 | 4 | *lithium 1000* |
| 21 | 32 | F | 17 | 13 | 15 | *lithium 600* |
| 22 | 46 | F | 30 | 9 | 0 | *lithium 500* |
| 23 | 38 | F | 15 | 7 | 2 | *lithium 800, quetiapine 500, flunitrazepam 2, magnesium oxide 1000, lubiprostone 24, linaclotide 0.5* |
| 24 | 43 | M | 17 | 2 | 0 | *lithium 400* |
| 25 | 55 | F | 35 | 10 | 0 | *lithium 600, lamotrigine 200, lorazepam 1, flunitrazepam 2* |
| 26 | 46 | M | 6 | 9 | 7 | *lithium 800, zolpidem 10, levomepromazine 10, flunitrazepam 2* |
| 27 | 21 | F | 0 | 6 | 10 | *lithium 300, aripiprazole 3, suvorexant 20* |
| 28 | 35 | M | 12 | 3 | 0 | *lithium 400* |
| 29 | 40 | F | 20 | 11 | 0 | *lithium 1300, methylphenidate hydrochloride 27* |
| 30 | 39 | F | 2 | 10 | 2 | *lithium 800, brexpiprazole 1, ramelteon 8, clonazepam 0.5*, clonidine 75, |
| 31 | 48 | F | 8 | 9 | 13 | *sodium valproate 400, lamotrigine 200, eszopiclone 2, quetiapine 100, lomerizine hydrochloride 10, lorazepam 6,* |
| 32 | 39 | M | 4 | 3 | 12 | *lithium 1000, benzbromarone 100*, montelukast 10 |
| 33 | 56 | F | 16 | 13 | 5 | *lithium 1000, clonazepam 1, brotizolam 0.25, lorazepam 1.5, promethazine 25* |
| 34 | 59 | F | 19 | 9 | 0 | *lithium 400, lamotrigine 100, zolpidem 10, clonazepam 1, suvorexant 20, sodium valproate 400, nitrazepam 5, levothyroxine sodium hydrate 75* |
| 35 | 51 | F | 2 | 2 | 8 | *lithium 200, quetiapine 12.5, eszopiclone 2* |
| 36 | 49 | F | 24 | 0 | 24 | *lithium 100, flunitrazepam 1, sertraline 100, lemborexant 5, levomepromazine 5* |
| 37 | 38 | M | 20 | 0 | 12 | *lithium 200, quetiapine 50, olanzapine 5* |

M, male; F, female; DOI, duration of illness; HAMD-17, 17-item Hamilton Depression Rating Scale; YMRS, Young Mania Rating Scale

**Supplemental Table 3. Demographic and Clinical Characteristics of Patients with depression.**

| No. | Age, years | Sex | DOI, years | HAMD-17 total score | Medications prescribed, mg/day, Psychotropic drugs are written in italics |
| --- | --- | --- | --- | --- | --- |
| 1 | 44 | M | 7 | 15 | *sertraline 25* |
| 2 | 49 | M | 11 | 10 | *nortriptyline 100, loflazepate 1,* allopurinol 100 |
| 3 | 33 | M | 12 | 8 | *sertraline 100, nortriptyline 25, zolpidem 5, etizolam 1.5, alprazolam 1.2* |
| 4 | 39 | M | 7 | 10 | *sertraline 100, zolpidem 10* |
| 5 | 42 | M | 12 | 2 | *sertraline 75, brotizolam 0.5,* fexofenadine 120 |
| 6 | 47 | M | 2 | 2 | None |
| 7 | 40 | M | 2 | 0 | *duloxetine 40* |
| 8 | 33 | M | 2 | 8 | *duloxetine 60, aripiprazole 3, quetiapine 75, lorazepam 1, brotizolam 0.25, suvorexant 20* |
| 9 | 48 | M | 7 | 9 | *flunitrazepam 1* |
| 10 | 48 | M | 7 | 0 | sertraline 100 |
| 11 | 54 | F | 9 | 2 | *duloxetine 60* |
| 12 | 37 | M | 3 | 6 | *escitalopram10* |
| 13 | 58 | M | 0 | 20 | *sertraline 100, eszopiclone 3* |
| 14 | 38 | F | 18 | 4 | *sertraline 100, aripiprazole 3* |
| 15 | 56 | M | 1 | 8 | *sertraline 50, lemborexant 10* |
| 16 | 43 | F | 2 | 3 | *vortioxetine 10* |
| 17 | 41 | M | 0 | 25 | *duloxetine 60, mosapride citrate hydrate 5, eszopiclone 2* |
| 18 | 36 | M | 6 | 3 | *duloxetine 60, sertraline 100* |
| 19 | 55 | M | 1 | 5 | *mirtazapine 15, brotizolam 0.25* |
| 20 | 26 | M | 4 | 11 | *fluvoxamine maleate 125* |
| 21 | 43 | M | 0 | 16 | *duloxetine 40, lemborexant 10, lorazepam 1.5* |
| 22 | 50 | F | 17 | 7 | *venlafaxine 112.5, quetiapine 225, sennoside 12* |
| 23 | 43 | M | 6 | 18 | *lorazepam 1.5, sertraline 75, zolpidem 5, loxoprofen 60* |
| 24 | 49 | F | 29 | 13 | *amoxapine 150, clomipramine 225, brexpiprazole 1, methylphenidate 18, zolpidem 10,  triazolam 0.5, levomepromazine 45,　clonazepam 2* |
| 25 | 45 | F | 21 | 6 | *vortioxetine 20, duloxetine 60, amoxapine 75, cloxazolam 1,　lamotrigine 325, aripiprazole 3*,  sennoside 36, magnesium oxide 1500, pitavastatin calcium 1 |
| 26 | 37 | M | 20 | 15 | *sertraline 75, zolpidem 5, suvorexant 20, clomipramine 30,  clomipramine 30, lithium 800, thyronamine 1.5, clonazepam 0.5* |
| 27 | 46 | F | 1 | 13 | *venlafaxine 150, mirtazapine 15, aripiprazole 6* |
| 28 | 35 | M | 3 | 0 | None |
| 29 | 42 | M | 7 | 25 | *amoxapine 30, zolpidem 10* |
| 30 | 47 | M | 17 | 22 | *aripiprazole 6, lorazepam 0.5, lithium 400, nortriptyline 70, zolpidem 10* |
| 31 | 33 | M | 7 | 17 | *brotizolam 0.25, flunitrazepam 1, trazodon 25, vortioxetine 20, suvorexant 20, venlafaxine 150* |
| 32 | 30 | F | 6 | 20 | *flunitrazepam 2, ramelteon 8, venlafaxine 225* |
| 33 | 49 | M | 2 | 23 | *brotizolam 0.25, quetiapine 25, paroxetine hydrochloride hydrate 50,  risperidone 2, eperisone 0.5, amitriptyline 75* |
| 34 | 43 | M | 24 | 13 | None |
| 35 | 46 | M | 1 | 8 | *duloxetine 60, bromazepam 12, lithium 200* |

M, male; F, female; DOI, duration of illness; HAMD-17, 17-item Hamilton Depression Rating Scale

**Supplemental Table 4. Demographic and Clinical Characteristics of Patients with ASD.**

| No. | Age, years | Sex | ADOS-2 CSS | Medications prescribed, mg/day, Psychotropic drugs are written in italics |
| --- | --- | --- | --- | --- |
| 1 | 43 | M | 10 | *lithium 800, lorazepam 0.5, zolpidem 2.5, quetiapine 12.5* |
| 2 | 31 | M | 10 | *brotizolam 0.25, alprazolam 0.4* |
| 3 | 36 | M | 8 | *quetiapine 100, mirtazapine 100* |
| 4 | 33 | M | 10 | *methylphenidate hydrochloride 36, ramelteon 4, montelukast 10* |
| 5 | 36 | M | 10 | *zolpidem 10, flunitrazepam 1* |
| 6 | 41 | M | 9 | *amitriptyline 100, aripiprazole 1, carbamazepine 300, bromazepam 15* |
| 7 | 46 | M | 6 | *lithium 800, olanzapine 5, cloxazolam 5* |
| 8 | 47 | M | 8 | *sodium valproate 600, sertraline 75, brotizolam 0.25* |
| 9 | 30 | M | 9 | None |
| 10 | 44 | M | 10 | *bromazepam 2, mirtazapine 30, suvorexant 20, carbamazepine 100* |
| 11 | 38 | M | 8 | *eszopiclone 2*, ketoprofen 40 |
| 12 | 32 | M | 8 | *escitalopram 10, methylphenidate hydrochloride 45, quetiapine 600, sodium valproate 1200* |
| 13 | 29 | M | 4 | *aripiprazole 3, risperidone 1, atomoxetine 80, carbamazepine 500* |
| 14 | 37 | M | 8 | *sertraline 100, alprazolam 1.2* |
| 15 | 51 | M | 9 | *sertraline 100, zolpidem 10, etizolam 1.5, sulpiride 50*, polycarbophil calcium 6 |
| 16 | 22 | M | 6 | None |
| 17 | 24 | F | 7 | *escitalopram 10* |
| 18 | 31 | M | 3 | *sertraline 25, allopurinol 200*, lomerizine hydrochloride 10 |
| 19 | 46 | F | 3 | *suvorexant 15, trazodone 25, flunitrazepam 1, sulpiride 50*, metformin 50, dapagliflozin propylene glycolate hydrate 5, pitavastatin 1, levothyroxine sodium hydrate 100 |
| 20 | 33 | M | 7 | *amitriptyline 10* |
| 21 | 32 | F | 9 | *aripiprazole 3, suvorexant 15* |
| 22 | 34 | F | 2 | methylphenidate hydrochloride 60 |
| 23 | 29 | M | 5 | dried ferrous sulfate 105, mosapride 5 |
| 24 | 24 | M | 9 | *quetiapine 50, zolpidem 5* |
| 25 | 23 | M | 9 | *suvorexant 15* |
| 26 | 29 | M | 9 | mesalazine enteric coated tablets 2400 |
| 27 | 23 | M | 9 | *duloxetine 60* |
| 28 | 31 | M | 10 | None |
| 29 | 32 | M | 8 | None |
| 30 | 28 | M | 6 | *risperidone 1, paroxetine 37.5, febuxostat 10* |
| 31 | 31 | F | 6 | *lemborexant 2.5*, dienogest 1 |
| 32 | 25 | F | 5 | *brexpiprazole 1,* sodium ferrous citrate 100 |
| 33 | 37 | M | 7 | *aripiprazole 6, sodium valproate 600, flunitrazepam 2, quetiapine 100* |
| 34 | 28 | F | 6 | *ramelteon 8, lemborexant 10* |
| 35 | 24 | F | 4 | None |

M, male; F, female; ADOS-2 CSS, Autism Diagnostic Observation Schedule Second Edition calibrated severity score

**Supplemental Table 5. *BP*_ND_ and SUVR for each brain region in healthy participants.**

|  | *BP*_ND_ | SUVR_30-50min_WM | SUVR_30-50min_WB |
| --- | --- | --- | --- |
| Frontal cortex | 1.156 ± 0.196 | 2.096 ± 0.189 | 1.106 ± 0.037 |
| Parietal cortex | 1.143 ± 0.178 | 2.083 ± 0.170 | 1.099 ± 0.030 |
| Occipital cortex | 1.181 ± 0.178 | 2.108 ± 0.171 | 1.113 ± 0.036 |
| Temporal cortex | 1.011 ± 0.169 | 1.975 ± 0.164 | 1.042 ± 0.033 |
| Cingulate cortex | 1.327 ± 0.205 | 2.271 ± 0.199 | 1.198 ± 0.053 |
| Caudate | 0.844 ± 0.237 | 1.803 ± 0.233 | 0.949 ± 0.078 |
| Putamen | 1.385 ± 0.212 | 2.303 ± 0.207 | 1.217 ± 0.046 |
| Hippocampus | 0.806 ± 0.135 | 1.801 ± 0.132 | 0.951 ± 0.038 |
| Amygdala | 0.783 ± 0.140 | 1.779 ± 0.141 | 0.940 ± 0.035 |
| Cerebellum | 1.431 ± 0.196 | 2.403 ± 0.194 | 1.268 ± 0.053 |

*BP*_ND_, SUVR_30-50min_WM and SUVR_30-50min_WB indicate mean±SD.

**Supplemental Table 6. Regression analysis between SUVR_30-50min_WB and *BP*_ND_ in healthy participants.**

| Subject IDs | Correlation coefficient | p value | Regression line |
| --- | --- | --- | --- |
| 1 | 0.9912 | <0.0001 | Y = 0.4689*X + 0.5382 |
| 2 | 0.9879 | <0.0001 | Y = 0.5085*X + 0.5828 |
| 3 | 0.9943 | <0.0001 | Y = 0.5247*X + 0.5474 |
| 4 | 0.9936 | <0.0001 | Y = 0.4684*X + 0.5382 |
| 5 | 0.9953 | <0.0001 | Y = 0.4605*X + 0.4970 |
| 6 | 0.9864 | <0.0001 | Y = 0.4389*X + 0.5642 |
| 7 | 0.9925 | <0.0001 | Y = 0.4464*X + 0.5457 |
| 8 | 0.9975 | <0.0001 | Y = 0.4309*X + 0.4873 |
| 9 | 0.9985 | <0.0001 | Y = 0.4738*X + 0.5175 |
| 10 | 0.9966 | <0.0001 | Y = 0.4618*X + 0.5231 |
| 11 | 0.9922 | <0.0001 | Y = 0.4460*X + 0.5419 |
| 12 | 0.9927 | <0.0001 | Y = 0.4766*X + 0.5639 |
| 13 | 0.9989 | <0.0001 | Y = 0.4958*X + 0.5368 |
| 14 | 0.9939 | <0.0001 | Y = 0.4799*X + 0.5430 |
| 15 | 0.9990 | <0.0001 | Y = 0.4232*X + 0.4955 |
| 16 | 0.9949 | <0.0001 | Y = 0.4307*X + 0.5562 |
| 17 | 0.9933 | <0.0001 | Y = 0.4649*X + 0.5521 |
| 18 | 0.9946 | <0.0001 | Y = 0.4393*X + 0.4829 |
| 19 | 0.9931 | <0.0001 | Y = 0.4286*X + 0.5139 |
| 20 | 0.9989 | <0.0001 | Y = 0.4752*X + 0.5163 |
| 21 | 0.9942 | <0.0001 | Y = 0.5114*X + 0.5807 |
| 22 | 0.9965 | <0.0001 | Y = 0.5793*X + 0.6153 |
| 23 | 0.9958 | <0.0001 | Y = 0.5362*X + 0.5562 |
| 24 | 0.9974 | <0.0001 | Y = 0.4630*X + 0.5283 |
| 25 | 0.9965 | <0.0001 | Y = 0.5032*X + 0.5660 |
| 26 | 0.9920 | <0.0001 | Y = 0.4906*X + 0.5240 |
| 27 | 0.9986 | <0.0001 | Y = 0.5420*X + 0.5487 |
| 28 | 0.9996 | <0.0001 | Y = 0.4599*X + 0.4958 |
| 29 | 0.9869 | <0.0001 | Y = 0.4484*X + 0.4941 |
| 30 | 0.9979 | <0.0001 | Y = 0.4940*X + 0.5651 |
| 31 | 0.9969 | <0.0001 | Y = 0.5262*X + 0.5776 |
| 32 | 0.9979 | <0.0001 | Y = 0.4622*X + 0.5381 |
| 33 | 0.9958 | <0.0001 | Y = 0.5586*X + 0.6039 |
| 34 | 0.9931 | <0.0001 | Y = 0.4547*X + 0.5244 |
| 35 | 0.9930 | <0.0001 | Y = 0.5418*X + 0.5771 |
| 36 | 0.9991 | <0.0001 | Y = 0.4806*X + 0.5088 |
| 37 | 0.9877 | <0.0001 | Y = 0.4558*X + 0.5838 |
| 38 | 0.9974 | <0.0001 | Y = 0.4850*X + 0.4832 |
| 39 | 0.9990 | <0.0001 | Y = 0.4604*X + 0.4848 |
| 40 | 0.9956 | <0.0001 | Y = 0.4672*X + 0.5461 |
| 41 | 0.9828 | <0.0001 | Y = 0.4802*X + 0.5627 |
| 42 | 0.9949 | <0.0001 | Y = 0.5579*X + 0.5897 |
| 43 | 0.9956 | <0.0001 | Y = 0.5420*X + 0.5858 |
| 44 | 0.9991 | <0.0001 | Y = 0.5712*X + 0.5784 |
| 45 | 0.9985 | <0.0001 | Y = 0.4948*X + 0.5338 |
| 46 | 0.9968 | <0.0001 | Y = 0.5018*X + 0.5358 |
| 47 | 0.9949 | <0.0001 | Y = 0.5106*X + 0.5347 |
| 48 | 0.9983 | <0.0001 | Y = 0.5418*X + 0.5699 |
| 49 | 0.9900 | <0.0001 | Y = 0.4480*X + 0.5704 |
| 50 | 0.9773 | <0.0001 | Y = 0.4890*X + 0.5990 |
| 51 | 0.9975 | <0.0001 | Y = 0.5462*X + 0.5560 |
| 52 | 0.9966 | <0.0001 | Y = 0.5291*X + 0.5447 |
| 53 | 0.9935 | <0.0001 | Y = 0.4781*X + 0.5570 |
| 54 | 0.9958 | <0.0001 | Y = 0.4783*X + 0.5159 |
| 55 | 0.9981 | <0.0001 | Y = 0.5259*X + 0.5598 |
| 56 | 0.9893 | <0.0001 | Y = 0.5853*X + 0.6003 |
| 57 | 0.9951 | <0.0001 | Y = 0.4732*X + 0.5309 |
| 58 | 0.9934 | <0.0001 | Y = 0.4914*X + 0.5235 |
| 59 | 0.9988 | <0.0001 | Y = 0.4712*X + 0.5373 |
| 60 | 0.9924 | <0.0001 | Y = 0.4578*X + 0.5301 |
| 61 | 0.9898 | <0.0001 | Y = 0.5089*X + 0.6092 |
| 62 | 0.9960 | <0.0001 | Y = 0.5252*X + 0.5817 |
| 63 | 0.9987 | <0.0001 | Y = 0.5764*X + 0.6265 |
| 64 | 0.9957 | <0.0001 | Y = 0.5042*X + 0.5488 |
| 65 | 0.9974 | <0.0001 | Y = 0.5102*X + 0.5264 |
| 66 | 0.9972 | <0.0001 | Y = 0.5100*X + 0.5701 |
| 67 | 0.9974 | <0.0001 | Y = 0.5374*X + 0.5451 |
| 68 | 0.9956 | <0.0001 | Y = 0.5196*X + 0.5316 |
| 69 | 0.9985 | <0.0001 | Y = 0.5591*X + 0.5838 |
| 70 | 0.9920 | <0.0001 | Y = 0.5105*X + 0.5547 |

**Supplemental Table 7. The coefficient of variation of SUVR_30-50min_WM and SUVR_30-50min_WB in healthy participants and patients with psychiatric disorders.**

|  | Coefficient of variation | | | | | |
| --- | --- | --- | --- | --- | --- | --- |
|  | Healthy participants | | Schizophrenia | | Bipolar disorder | |
|  | SUVR_30-50min_WM | SUVR_30-50min_WB | SUVR_30-50min_WM | SUVR_30-50min_WB | SUVR_30-50min_WM | SUVR_30-50min_WB |
| Frontal cortex | 0.090 | 0.034 | 0.091 | 0.030 | 0.076 | 0.024 |
| Parietal cortex | 0.082 | 0.028 | 0.095 | 0.038 | 0.075 | 0.030 |
| Occipital cortex | 0.081 | 0.032 | 0.089 | 0.035 | 0.071 | 0.029 |
| Temporal cortex | 0.083 | 0.031 | 0.094 | 0.031 | 0.072 | 0.020 |
| Cingulate cortex | 0.088 | 0.044 | 0.085 | 0.044 | 0.073 | 0.031 |
| Caudate | 0.129 | 0.082 | 0.146 | 0.105 | 0.115 | 0.065 |
| Putamen | 0.090 | 0.038 | 0.074 | 0.041 | 0.070 | 0.038 |
| Hippocampus | 0.073 | 0.040 | 0.080 | 0.036 | 0.081 | 0.039 |
| Amygdala | 0.079 | 0.037 | 0.082 | 0.034 | 0.084 | 0.041 |
| Cerebellum | 0.081 | 0.042 | 0.099 | 0.044 | 0.076 | 0.041 |

|  | Coefficient of variation | | | |
| --- | --- | --- | --- | --- |
|  | Depression | | ASD | |
|  | SUVR_30-50min_WM | SUVR_30-50min_WB | SUVR_30-50min_WM | SUVR_30-50min_WB |
| Frontal cortex | 0.092 | 0.028 | 0.083 | 0.025 |
| Parietal cortex | 0.088 | 0.028 | 0.085 | 0.025 |
| Occipital cortex | 0.089 | 0.028 | 0.073 | 0.027 |
| Temporal cortex | 0.078 | 0.018 | 0.085 | 0.023 |
| Cingulate cortex | 0.087 | 0.028 | 0.075 | 0.028 |
| Caudate | 0.127 | 0.074 | 0.109 | 0.060 |
| Putamen | 0.083 | 0.038 | 0.079 | 0.040 |
| Hippocampus | 0.083 | 0.039 | 0.082 | 0.031 |
| Amygdala | 0.071 | 0.033 | 0.082 | 0.032 |
| Cerebellum | 0.089 | 0.039 | 0.092 | 0.042 |

**Supplemental Table 8. Regression analysis between SUVR_30-50min_WB and *BP*_ND_ in patients with schizophrenia.**

| Subject IDs | Correlation coefficient | p value | Regression line |
| --- | --- | --- | --- |
| 1 | 0.9946 | <0.0001 | Y = 0.5485*X + 0.6110 |
| 2 | 0.9937 | <0.0001 | Y = 0.5395*X + 0.5700 |
| 3 | 0.9984 | <0.0001 | Y = 0.5679*X + 0.5785 |
| 4 | 0.9989 | <0.0001 | Y = 0.6075*X + 0.6317 |
| 5 | 0.9960 | <0.0001 | Y = 0.5877*X + 0.6170 |
| 6 | 0.9981 | <0.0001 | Y = 0.5618*X + 0.5868 |
| 7 | 0.9985 | <0.0001 | Y = 0.5612*X + 0.5699 |
| 8 | 0.9970 | <0.0001 | Y = 0.5232*X + 0.5918 |
| 9 | 0.9956 | <0.0001 | Y = 0.6449*X + 0.6610 |
| 10 | 0.9993 | <0.0001 | Y = 0.5376*X + 0.5501 |
| 11 | 0.9977 | <0.0001 | Y = 0.5485*X + 0.6110 |
| 12 | 0.9897 | <0.0001 | Y = 0.5814*X + 0.6028 |
| 13 | 0.9934 | <0.0001 | Y = 0.5200*X + 0.5833 |
| 14 | 0.9969 | <0.0001 | Y = 0.5970*X + 0.5983 |
| 15 | 0.9898 | <0.0001 | Y = 0.4673*X + 0.5619 |
| 16 | 0.9944 | <0.0001 | Y = 0.5125*X + 0.5819 |
| 17 | 0.9965 | <0.0001 | Y = 0.5232*X + 0.5730 |
| 18 | 0.9941 | <0.0001 | Y = 0.4679*X + 0.5779 |
| 19 | 0.9950 | <0.0001 | Y = 0.4992*X + 0.5300 |
| 20 | 0.9990 | <0.0001 | Y = 0.6035*X + 0.6291 |
| 21 | 0.9987 | <0.0001 | Y = 0.5188*X + 0.5518 |
| 22 | 0.9965 | <0.0001 | Y = 0.4649*X + 0.5601 |
| 23 | 0.9778 | <0.0001 | Y = 0.4951*X + 0.6060 |
| 24 | 0.9996 | <0.0001 | Y = 0.5930*X + 0.6293 |
| 25 | 0.9942 | <0.0001 | Y = 0.4802*X + 0.5406 |
| 26 | 0.9985 | <0.0001 | Y = 0.4852*X + 0.5481 |
| 27 | 0.9891 | <0.0001 | Y = 0.5203*X + 0.5990 |
| 28 | 0.9984 | <0.0001 | Y = 0.5550*X + 0.5762 |
| 29 | 0.9923 | <0.0001 | Y = 0.5091*X + 0.5705 |
| 30 | 0.9898 | <0.0001 | Y = 0.5116*X + 0.5690 |
| 31 | 0.9971 | <0.0001 | Y = 0.5992*X + 0.6231 |
| 32 | 0.9970 | <0.0001 | Y = 0.5568*X + 0.6306 |
| 33 | 0.9962 | <0.0001 | Y = 0.4824*X + 0.5392 |
| 34 | 0.9959 | <0.0001 | Y = 0.6414*X + 0.6262 |
| 35 | 0.9955 | <0.0001 | Y = 0.5916*X + 0.6287 |
| 36 | 0.9940 | <0.0001 | Y = 0.4679*X + 0.5185 |
| 37 | 0.9953 | <0.0001 | Y = 0.6212*X + 0.6347 |
| 38 | 0.9988 | <0.0001 | Y = 0.4913*X + 0.5298 |
| 39 | 0.9929 | <0.0001 | Y = 0.5019*X + 0.5566 |
| 40 | 0.9951 | <0.0001 | Y = 0.4992*X + 0.6122 |
| 41 | 0.9962 | <0.0001 | Y = 0.5222*X + 0.5344 |
| 42 | 0.9955 | <0.0001 | Y = 0.4798*X + 0.5522 |

**Supplemental Table 9. Regression analysis between SUVR_30-50min_WB and *BP*_ND_ in patients with bipolar disorder.**

| Subject IDs | Correlation coefficient | p value | Regression line |
| --- | --- | --- | --- |
| 1 | 0.9953 | <0.0001 | Y = 0.5252*X + 0.5604 |
| 2 | 0.9969 | <0.0001 | Y = 0.6145*X + 0.6566 |
| 3 | 0.9967 | <0.0001 | Y = 0.5631*X + 0.5447 |
| 4 | 0.9894 | <0.0001 | Y = 0.4718*X + 0.5471 |
| 5 | 0.9987 | <0.0001 | Y = 0.5250*X + 0.5560 |
| 6 | 0.9960 | <0.0001 | Y = 0.5015*X + 0.5734 |
| 7 | 0.9989 | <0.0001 | Y = 0.5683*X + 0.5947 |
| 8 | 0.9968 | <0.0001 | Y = 0.5399*X + 0.5828 |
| 9 | 0.9989 | <0.0001 | Y = 0.5392*X + 0.5557 |
| 10 | 0.9975 | <0.0001 | Y = 0.5077*X + 0.5665 |
| 11 | 0.9989 | <0.0001 | Y = 0.5949*X + 0.6067 |
| 12 | 0.9950 | <0.0001 | Y = 0.5543*X + 0.5985 |
| 13 | 0.9951 | <0.0001 | Y = 0.4604*X + 0.5607 |
| 14 | 0.9984 | <0.0001 | Y = 0.5747*X + 0.5962 |
| 15 | 0.9968 | <0.0001 | Y = 0.5456*X + 0.5946 |
| 16 | 0.9948 | <0.0001 | Y = 0.4488*X + 0.5185 |
| 17 | 0.9979 | <0.0001 | Y = 0.5071*X + 0.5336 |
| 18 | 0.9963 | <0.0001 | Y = 0.5048*X + 0.5680 |
| 19 | 0.9992 | <0.0001 | Y = 0.5322*X + 0.5673 |
| 20 | 0.9967 | <0.0001 | Y = 0.4597*X + 0.5263 |
| 21 | 0.9980 | <0.0001 | Y = 0.5014*X + 0.5737 |
| 22 | 0.9934 | <0.0001 | Y = 0.5579*X + 0.5928 |
| 23 | 0.9902 | <0.0001 | Y = 0.5380*X + 0.5813 |
| 24 | 0.9979 | <0.0001 | Y = 0.4717*X + 0.5612 |
| 25 | 0.9948 | <0.0001 | Y = 0.4797*X + 0.5529 |
| 26 | 0.9966 | <0.0001 | Y = 0.5593*X + 0.6320 |
| 27 | 0.9961 | <0.0001 | Y = 0.5038*X + 0.5962 |
| 28 | 0.9972 | <0.0001 | Y = 0.4596*X + 0.5364 |
| 29 | 0.9946 | <0.0001 | Y = 0.4859*X + 0.5155 |
| 30 | 0.9953 | <0.0001 | Y = 0.5320*X + 0.6078 |
| 31 | 0.9969 | <0.0001 | Y = 0.5833*X + 0.5972 |
| 32 | 0.9745 | <0.0001 | Y = 0.4538*X + 0.5740 |
| 33 | 0.9945 | <0.0001 | Y = 0.5109*X + 0.5669 |
| 34 | 0.9932 | <0.0001 | Y = 0.5271*X + 0.5960 |
| 35 | 0.9983 | <0.0001 | Y = 0.4890*X + 0.5196 |
| 36 | 0.9972 | <0.0001 | Y = 0.5297*X + 0.5562 |
| 37 | 0.9764 | <0.0001 | Y = 0.4448*X + 0.5711 |

**Supplemental Table 10. Regression analysis between SUVR_30-50min_WB and *BP*_ND_ in patients with depression.**

| Subject IDs | Correlation coefficient | p value | Regression line |
| --- | --- | --- | --- |
| 1 | 0.9957 | <0.0001 | Y = 0.5650*X + 0.5900 |
| 2 | 0.9972 | <0.0001 | Y = 0.5667*X + 0.6058 |
| 3 | 0.9974 | <0.0001 | Y = 0.5165*X + 0.5390 |
| 4 | 0.9989 | <0.0001 | Y = 0.6214*X + 0.6226 |
| 5 | 0.9984 | <0.0001 | Y = 0.5225*X + 0.5602 |
| 6 | 0.9888 | <0.0001 | Y = 0.5562*X + 0.6023 |
| 7 | 0.9954 | <0.0001 | Y = 0.5365*X + 0.5488 |
| 8 | 0.9966 | <0.0001 | Y = 0.5706*X + 0.6032 |
| 9 | 0.9990 | <0.0001 | Y = 0.5388*X + 0.5650 |
| 10 | 0.9979 | <0.0001 | Y = 0.5005*X + 0.5353 |
| 11 | 0.9900 | <0.0001 | Y = 0.4970*X + 0.6140 |
| 12 | 0.9984 | <0.0001 | Y = 0.4450*X + 0.4951 |
| 13 | 0.9950 | <0.0001 | Y = 0.5383*X + 0.6068 |
| 14 | 0.9947 | <0.0001 | Y = 0.4960*X + 0.5632 |
| 15 | 0.9938 | <0.0001 | Y = 0.5553*X + 0.6023 |
| 16 | 0.9930 | <0.0001 | Y = 0.4983*X + 0.5758 |
| 17 | 0.9942 | <0.0001 | Y = 0.4906*X + 0.5047 |
| 18 | 0.9985 | <0.0001 | Y = 0.4987*X + 0.5210 |
| 19 | 0.9965 | <0.0001 | Y = 0.6129*X + 0.6126 |
| 20 | 0.9977 | <0.0001 | Y = 0.4815*X + 0.4947 |
| 21 | 0.9957 | <0.0001 | Y = 0.5052*X + 0.5384 |
| 22 | 0.9936 | <0.0001 | Y = 0.4655*X + 0.4761 |
| 23 | 0.9925 | <0.0001 | Y = 0.5099*X + 0.5660 |
| 24 | 0.9948 | <0.0001 | Y = 0.5474*X + 0.5919 |
| 25 | 0.9968 | <0.0001 | Y = 0.5144*X + 0.5386 |
| 26 | 0.9975 | <0.0001 | Y = 0.4719*X + 0.5183 |
| 27 | 0.9937 | <0.0001 | Y = 0.5354*X + 0.5623 |
| 28 | 0.9956 | <0.0001 | Y = 0.4906*X + 0.5324 |
| 29 | 0.9932 | <0.0001 | Y = 0.5170*X + 0.5578 |
| 30 | 0.9969 | <0.0001 | Y = 0.5517*X + 0.5584 |
| 31 | 0.9954 | <0.0001 | Y = 0.4740*X + 0.5323 |
| 32 | 0.9990 | <0.0001 | Y = 0.4672*X + 0.4907 |
| 33 | 0.9962 | <0.0001 | Y = 0.5174*X + 0.5512 |
| 34 | 0.9923 | <0.0001 | Y = 0.5109*X + 0.5689 |
| 35 | 0.9823 | <0.0001 | Y = 0.5163*X + 0.5980 |

**Supplemental Table 11. Regression analysis between SUVR_30-50min_WB and *BP*_ND_ in patients with ASD.**

| Subject IDs | Correlation coefficient | p value | Regression line |
| --- | --- | --- | --- |
| 1 | 0.9994 | <0.0001 | Y = 0.5653*X + 0.5941 |
| 2 | 0.9934 | <0.0001 | Y = 0.4672*X + 0.5632 |
| 3 | 0.9947 | <0.0001 | Y = 0.5255*X + 0.6004 |
| 4 | 0.9976 | <0.0001 | Y = 0.5398*X + 0.5826 |
| 5 | 0.9969 | <0.0001 | Y = 0.5583*X + 0.5767 |
| 6 | 0.9969 | <0.0001 | Y = 0.5797*X + 0.5909 |
| 7 | 0.9977 | <0.0001 | Y = 0.5658*X + 0.6020 |
| 8 | 0.9988 | <0.0001 | Y = 0.5810*X + 0.6140 |
| 9 | 0.9972 | <0.0001 | Y = 0.5089*X + 0.5502 |
| 10 | 0.9959 | <0.0001 | Y = 0.5706*X + 0.6211 |
| 11 | 0.9921 | <0.0001 | Y = 0.5448*X + 0.5719 |
| 12 | 0.9916 | <0.0001 | Y = 0.5295*X + 0.5704 |
| 13 | 0.9973 | <0.0001 | Y = 0.4795*X + 0.5312 |
| 14 | 0.9959 | <0.0001 | Y = 0.5107*X + 0.5519 |
| 15 | 0.9940 | <0.0001 | Y = 0.5401*X + 0.6440 |
| 16 | 0.9967 | <0.0001 | Y = 0.4519*X + 0.5054 |
| 17 | 0.9976 | <0.0001 | Y = 0.5371*X + 0.5930 |
| 18 | 0.9940 | <0.0001 | Y = 0.4624*X + 0.5213 |
| 19 | 0.9988 | <0.0001 | Y = 0.5692*X + 0.5947 |
| 20 | 0.9940 | <0.0001 | Y = 0.5380*X + 0.5941 |
| 21 | 0.9952 | <0.0001 | Y = 0.5604*X + 0.5841 |
| 22 | 0.9963 | <0.0001 | Y = 0.5524*X + 0.6126 |
| 23 | 0.9860 | <0.0001 | Y = 0.4937*X + 0.5653 |
| 24 | 0.9690 | <0.0001 | Y = 0.4439*X + 0.5046 |
| 25 | 0.9934 | <0.0001 | Y = 0.5131*X + 0.5718 |
| 26 | 0.9956 | <0.0001 | Y = 0.4517*X + 0.4913 |
| 27 | 0.9981 | <0.0001 | Y = 0.5099*X + 0.5262 |
| 28 | 0.9923 | <0.0001 | Y = 0.4744*X + 0.5687 |
| 29 | 0.9888 | <0.0001 | Y = 0.5105*X + 0.5953 |
| 30 | 0.9935 | <0.0001 | Y = 0.4447*X + 0.5396 |
| 31 | 0.9905 | <0.0001 | Y = 0.4707*X + 0.5477 |
| 32 | 0.9936 | <0.0001 | Y = 0.4567*X + 0.5500 |
| 33 | 0.9912 | <0.0001 | Y = 0.4530*X + 0.5043 |
| 34 | 0.9948 | <0.0001 | Y = 0.4481*X + 0.5246 |
| 35 | 0.9942 | <0.0001 | Y = 0.4485*X + 0.5318 |

**References.**

1. American Psychiatric Association. *Diagnostic and Statistical Manual of Mental Disorders, 4th ed.* American Psychiatric Association: Washington, D.C., 1994.

2. First MB, Spitzer RL, Gibbon M, Williams JBW. *Structured clinical interview for DSM-IV axis I disorders, clinician version (SCID-CV)*. American Psychiatric Press: Washington, D.C., 1996.

3. American Psychiatric Association. *Diagnostic and statistical manual of mental disorders (5th Ed)*. American Psychiatric Publishing: Arlington, VA, 2013.

4. World Health Organization. *The ICD-10 classification of mental and behavioural disorders: clinical descriptions and diagnostic guidelines*. WHO: Geneva, 1992.

5. Okazawa H, Ikawa M, Jung M, Maruyama R, Tsujikawa T, Mori T *et al.* Multimodal analysis using [(11)C]PiB-PET/MRI for functional evaluation of patients with Alzheimer's disease. *EJNMMI Res* 2020; **10**(1)**:** 30.

6. Hammers A, Allom R, Koepp MJ, Free SL, Myers R, Lemieux L *et al.* Three-dimensional maximum probability atlas of the human brain, with particular reference to the temporal lobe. *Hum Brain Mapp* 2003; **19**(4)**:** 224-247.

7. Miyazaki T, Nakajima W, Hatano M, Shibata Y, Kuroki Y, Arisawa T *et al.* Visualization of AMPA receptors in living human brain with positron emission tomography. *Nat Med* 2020; **26**(2)**:** 281-288.

8. Ashburner J. A fast diffeomorphic image registration algorithm. *Neuroimage* 2007; **38**(1)**:** 95-113.

9. Ashburner J, Friston KJ. Unified segmentation. *Neuroimage* 2005; **26**(3)**:** 839-851.

10. Avants BB, Epstein CL, Grossman M, Gee JC. Symmetric diffeomorphic image registration with cross-correlation: evaluating automated labeling of elderly and neurodegenerative brain. *Med Image Anal* 2008; **12**(1)**:** 26-41.

11. Klein A, Andersson J, Ardekani BA, Ashburner J, Avants B, Chiang MC *et al.* Evaluation of 14 nonlinear deformation algorithms applied to human brain MRI registration. *Neuroimage* 2009; **46**(3)**:** 786-802.

12. Tustison NJ, Cook PA, Klein A, Song G, Das SR, Duda JT *et al.* Large-scale evaluation of ANTs and FreeSurfer cortical thickness measurements. *Neuroimage* 2014; **99:** 166-179.

13. Standard PET imaging protocols and phantom test procedures and criteria: executive summary. 2017, Accessed Date Accessed 2017 Accessed.
